# Cross-Dataset Generalization of Deep Learning-Based Detectors for Intracranial Hemorrhage Subtype Localization on Noncontrast Head CT: A Comparative Study

**DOI:** 10.3390/diagnostics16111705

**Published:** 2026-06-02

**Authors:** Chiao-Hua Lee, Hikam Muzakky, Cheng-En Juan, Chia-Ching Chang, Ya-Hui Li, Tung-Yang Lee, Cheng-Hsuan Juan, Ming-Ting Tsai, Chun-Jung Juan

**Affiliations:** 1 Department of Medical Imaging, China Medical University Hsinchu Hospital, Hsinchu 302, Taiwan; 2 China Medical University Hospital, Taichung 404, Taiwan; 3 Master’s Program of Biomedical Informatics and Biomedical Engineering, Feng Chia University, Taichung 407, Taiwan; 4 Department of Management Science, National Yang Ming Chiao Tung University, Hsinchu 300, Taiwan; 5 Graduate Institute of Biomedical Electronics and Bioinformatics, National Taiwan University, Taipei 106, Taiwan; 6 Show Chwan Memorial Hospital, Changhua 500, Taiwan; 7 Department of Medical Imaging, China Medical University Hospital, Taichung 404, Taiwan; 8 Department of Radiology, School of Medicine, College of Medicine, China Medical University, Taichung 406, Taiwan; 9 Department of Biomedical Engineering and Environmental Sciences, National Tsing Hua University, Hsinchu 300, Taiwan; 10 Department of Computer Science and Information Engineering, National Taiwan University, Taipei 106, Taiwan

**Keywords:** intracranial hemorrhage, noncontrast head CT, deep learning, object localization

## Abstract

**Background/Objectives:** To evaluate the effect of detector architecture and dataset characteristics on intracranial hemorrhage (ICH) subtype localization on noncontrast head CT, with emphasis on bidirectional cross-dataset generalization. **Methods:** This retrospective study analyzed two publicly available datasets: the Brain Hemorrhage Extended (BHX) dataset and the RSNA 2019+ dataset. Models were trained and internally validated on one dataset and externally tested on the other dataset in both directions: BHX-to-RSNA+ and RSNA+-to-BHX. Six representative deep learning detectors, including CNN-based one-stage and two-stage detectors and a Swin Transformer-based RT-DETR (Swin-RT-DETR) variant, were evaluated. Localization performance was assessed using mean average precision at a bounding-box intersection-over-union threshold of 0.5 (mAP@50), bounding-box Dice similarity coefficient (BB-DSC), and bounding-box intersection-over-union (BB-IoU). Image-level and patient-level analyses were performed, with Bonferroni correction applied for statistical comparisons. Dataset characterization analyses were performed to compare subtype prevalence, bounding-box geometry, lesion burden, annotation density, and spatial distribution. **Results:** Under internal validation, Swin-RT-DETR achieved competitive or superior performance across several ICH subtypes, but its advantage was subtype-dependent rather than uniform. Faster R-CNN with a ResNeXt101 backbone achieved comparable IVH performance and higher IPH BB-DSC and BB-IoU, whereas Swin-RT-DETR performed better for SAH, SDH, and EDH. External validation showed substantial performance degradation across architectures, subtypes, and validation directions. Absolute BB-DSC reductions for Swin-RT-DETR ranged from approximately 0.54–0.79 in the BHX-to-RSNA+ direction and 0.17–0.74 in the RSNA+-to-BHX direction. Similar degradation patterns were observed at the patient level. Statistical comparisons showed fewer significant model-level differences under external validation, suggesting attenuation of architecture-specific advantages under domain shift. Dataset characterization analysis demonstrated differences in subtype distribution, bounding-box geometry, lesion burden, annotation density, and spatial localization patterns between BHX and RSNA+. **Conclusions:** ICH subtype localization performance is strongly influenced by dataset characteristics, annotation heterogeneity, and domain shift. Although Transformer-based hierarchical feature extraction showed subtype-dependent advantages under internal validation, these advantages diminished under bidirectional external validation. These findings highlight the need for dataset characterization, external validation, patient-level evaluation, and task-specific clinical benchmarks before automated ICH localization models can be considered for real-world clinical integration.

## 1. Introduction

Intracranial hemorrhage (ICH) is a neurological emergency for which timely and accurate diagnosis is critical for patient management and treatment planning [1,2]. Deep learning studies have shown strong performance for automated ICH localization and subtype classification on noncontrast head CT, with several studies reporting area under the receiver operating characteristic curve (AUC) values exceeding 0.90 in internal and external validation cohorts [3,4,5]. However, image-level classification performance does not necessarily translate into clinically actionable localization, because a model may correctly predict the presence of hemorrhage without accurately identifying its anatomical extent or spatial distribution [6,7]. Accurate localization is clinically relevant because hemorrhage subtype, location, and distribution are closely related to etiology, management strategy, and prognosis. For example, intraparenchymal hemorrhage (IPH) and intraventricular hemorrhage (IVH) often present as relatively compact lesions, whereas subarachnoid hemorrhage (SAH) more often appears as irregular, diffuse, or multifocal hyperdensities along sulci and cisterns, and subdural hemorrhage (SDH) typically shows an elongated or crescentic extra-axial configuration along the cerebral convexity. These morphological differences create substantial challenges for automated localization, particularly when rectangular bounding boxes are used to represent irregular or spatially diffuse hemorrhage patterns [8].

Several object localization frameworks have been applied to ICH localization to bridge the gap between image-level classification and spatially explicit prediction. Prior studies have primarily used convolutional neural network (CNN)-based one-stage detectors, such as YOLO variants and RetinaNet, or two-stage detectors, such as Faster R-CNN [9,10,11,12,13,14,15]. Additional CNN-based approaches have explored 3D convolutional architectures for ICH classification with varying dataset sizes [16], efficient segmentation pipelines using lightweight CNN architectures [17], and multi-channel pseudo-color input strategies to enhance hemorrhage detection [18]. Recent work has also evaluated commercially available AI tools for post-traumatic ICH detection in clinical settings [19] and explored the diagnostic potential of large language model–based approaches for ICH identification [20]. Despite these advances, most studies have relied on internal validation, leaving cross-dataset robustness insufficiently characterized.

Segmentation-based approaches may provide more anatomically precise lesion delineation than bounding-box detection, especially for thin, diffuse, or irregular hemorrhage patterns. However, segmentation frameworks require pixel-level annotations [21], which are not consistently available across the public datasets used in this study. Therefore, we focused on bounding-box detection to enable controlled cross-dataset comparison using harmonized annotation formats. More recently, Transformer-based and hybrid CNN–Transformer architectures have attracted attention in medical imaging because of their capacity for contextual modeling and multiscale feature representation. Nevertheless, their role in ICH subtype localization, especially under external validation, remains insufficiently characterized. An additional limitation of the existing literature is that many ICH localization studies have relied on single-center or single-source datasets with internal train–test splits. Such validation designs may overestimate performance when training and testing images share similar acquisition protocols, annotation conventions, subtype distributions, and lesion morphology. In clinical deployment, however, models are expected to generalize across institutions, scanners, patient populations, and labeling practices. Therefore, it remains unclear whether improved detector architecture alone is sufficient to achieve robust ICH localization, or whether dataset characteristics and annotation heterogeneity exert a larger influence on external performance.

We hypothesized that dataset characteristics, annotation geometry, and hemorrhage morphology substantially influence external localization robustness, particularly for rare, thin, diffuse, or irregular hemorrhage subtypes. In this study, we systematically compared CNN-based one-stage detectors, CNN-based two-stage detectors, and a Swin Transformer-based RT-DETR variant for ICH subtype localization on noncontrast head CT. The goal was not only to compare detector architectures, but also to evaluate how localization performance changes under cross-dataset validation. To test this hypothesis, we performed internal and external validation using the BHX and RSNA+ datasets, together with dataset characterization analyses to examine subtype prevalence, bounding-box geometry, lesion burden, annotation density, and spatial distribution.

## 2. Materials and Methods

### 2.1. Datasets

**Figure 1:**
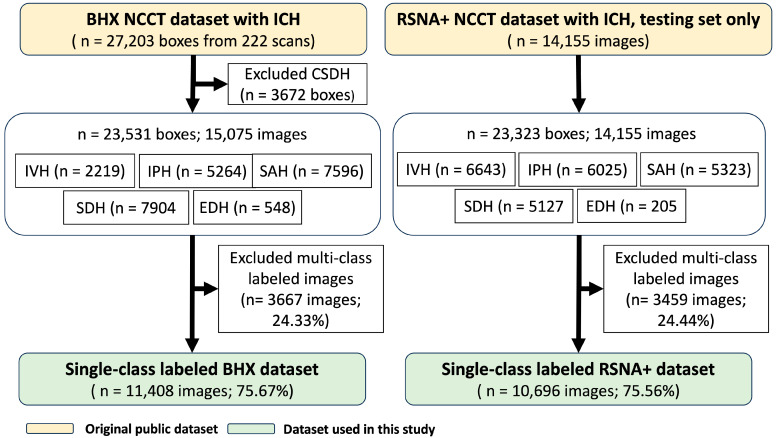
Dataset composition and case selection. Single-class labeled images were included for controlled subtype-specific comparison, whereas chronic subdural hematoma (CSDH) and multi-class labeled images were excluded. The final single-class BHX and RSNA+ datasets were used for bidirectional cross-dataset validation, including internal train/test splitting and reciprocal external testing. Multi-class labeled images accounted for 24.33% of BHX and 24.44% of RSNA+. NCCT indicates noncontrast computed tomography; ICH, intracranial hemorrhage; IVH, intraventricular hemorrhage; IPH, intraparenchymal hemorrhage; SAH, subarachnoid hemorrhage; SDH, subdural hemorrhage; EDH, epidural hemorrhage; and CSDH, chronic subdural hemorrhage.

This retrospective study analyzed de-identified noncontrast head CT scans from two publicly available datasets: the RSNA 2019+ dataset [14] and the Brain Hemorrhage Extended (BHX) dataset [22] (Figure 1). Because only de-identified publicly available datasets were used, this study was exempt from ethics committee review and from the requirement for informed consent according to the official government notices issued by the Department of Health, Executive Yuan, Taiwan [23,24]. Detailed dataset characteristics, inclusion criteria, and annotation procedures are provided in Supplementary S1 and Table S1.

The RSNA 2019+ dataset was derived from the RSNA 2019 Brain Hemorrhage Challenge 2019 [25]. The RSNA test set consisted of 14,155 slices, of which 10,696 single-class labeled images from 1273 patients were selected for the present study, accounting for 75.56% of the original RSNA+ dataset. Multi-class labeled images accounted for 24.44% of the RSNA+ test set.

The BHX dataset was derived from the original CQ500 cohort, which includes 491 patients acquired at six centers in New Delhi, India. After exclusion of chronic subdural hemorrhage, the BHX dataset comprised 15,075 images with 23,531 annotated hemorrhage instances, including intraparenchymal hemorrhage (IPH), intraventricular hemorrhage (IVH), subarachnoid hemorrhage (SAH), subdural hemorrhage (SDH), and epidural hemorrhage (EDH). For the present study, 11,408 single-class labeled images were selected, accounting for 75.67% of the BHX dataset. Multi-class labeled images accounted for 24.33% of the BHX dataset.

Only single-class labeled images were included to reduce label ambiguity and allow consistent subtype-specific comparisons across datasets and architectures. Multi-class labeled images were excluded from model training and evaluation because coexisting hemorrhage subtypes may produce overlapping or adjacent bounding boxes, complicating subtype-specific attribution of localization errors. This single-class design was therefore used as a controlled experimental simplification, although it does not fully reflect the coexistence of hemorrhage subtypes encountered in clinical practice.

### 2.2. Study Design

**Figure 2:**
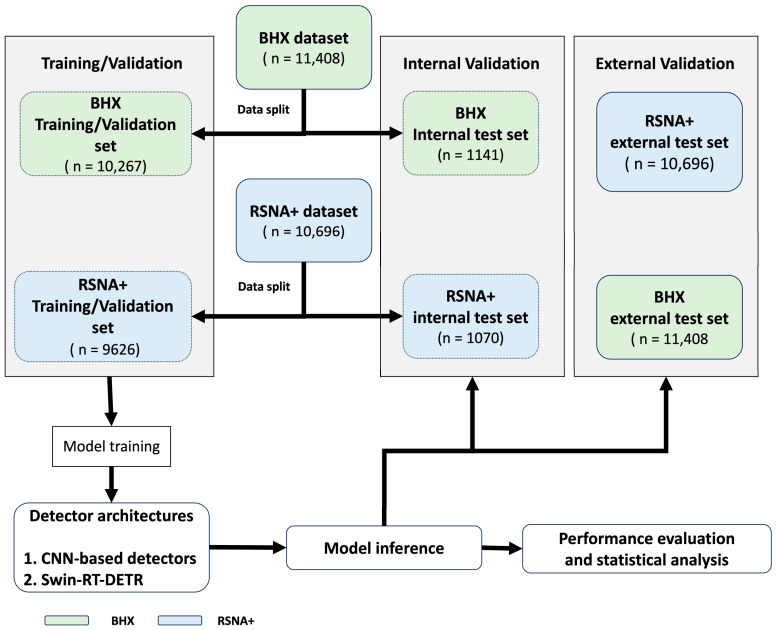
Study workflow and bidirectional cross-dataset validation design. Models were trained on BHX or RSNA+ training/validation set, internally tested on the corresponding held-out test set, and externally tested on the other dataset.

The overall study workflow, including dataset preparation, model training, internal validation, and bidirectional external validation, is illustrated in Figure 2. This study used a bidirectional cross-dataset validation design to evaluate both model performance and the symmetry of cross-dataset generalization.

In the first direction, the BHX single-class dataset was split into a training and validation set (n = 10,267 images) and an internal test set (n = 1141 images). Models were trained and optimized using the BHX training and validation set, internally evaluated on the BHX test set, and externally tested on the independent RSNA+ single-class test set (n = 10,696 images). In the reciprocal direction, the RSNA+ single-class dataset was split into a training and validation set (n = 9626 images) and an internal test set (n = 1070 images). The same model architectures were trained and optimized using the RSNA+ training and validation set, internally evaluated on the RSNA+ test set, and externally tested on the BHX dataset (n = 11,408 images).

In both directions, the external test dataset was used only for final external validation and was not used for model selection, hyperparameter tuning, or early stopping. Model performance was evaluated using mean average precision at a bounding-box intersection over union threshold of 0.5 (mAP@50), bounding-box Dice similarity coefficient (BB-DSC), and bounding-box intersection over union (BB-IoU). Image-level and patient-level analyses were performed to evaluate localization performance under internal and bidirectional external validation settings. Detailed statistical procedures are described in Section 2.7.

### 2.3. Image Preprocessing

All noncontrast CT images were converted into three standardized window settings: brain window (window width [WW], 80; window level [WL], 40), subdural window (WW, 50; WL, 175), and bone window (WW, 3000; WL, 500). These three windowed images were combined into a three-channel input to incorporate complementary tissue contrast information. Background removal was performed using fixed thresholding, morphological operations, and largest connected component extraction to isolate the head region. Details of image preprocessing are provided in Supplementary S2 (Figure S1).

### 2.4. Localization Models

**Figure 3:**
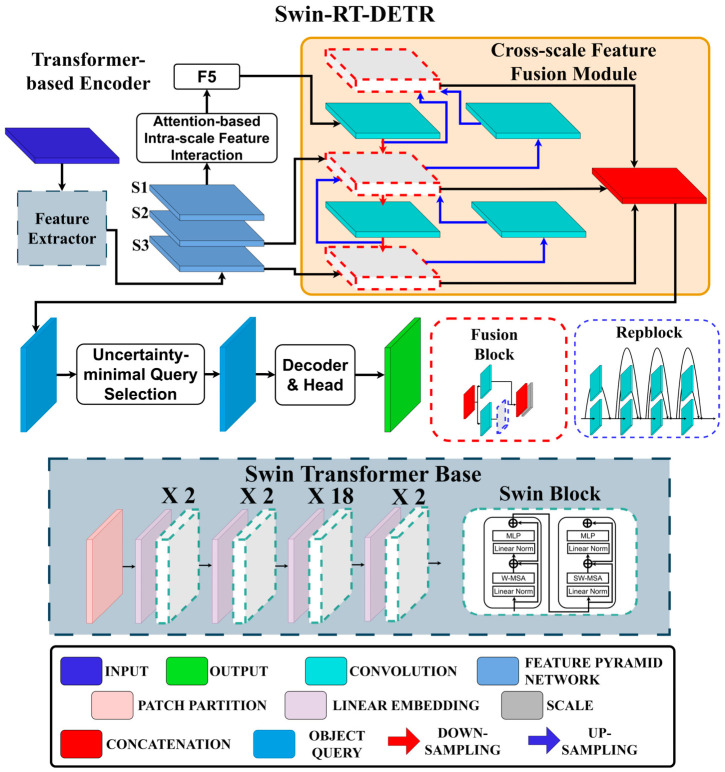
Swin Transformer–based RT-DETR variant used for ICH bounding-box localization. The original HGNetV2 backbone of RT-DETR was replaced with a Swin Transformer Base backbone, while the remaining RT-DETR detection framework was preserved. Orange box denotes cross-scale feature fusion module, red dashed box denotes fusion block, blue dashed box denotes reparameterization block and black dashed box denotes Swin Transformer Encoder.

To evaluate the potential contribution of Transformer-based feature extraction to ICH bounding-box localization, we implemented a Swin Transformer–based RT-DETR variant, referred to as Swin-RT-DETR (Figure 3). In this variant, the original HGNetV2 backbone of RT-DETR was replaced with a Swin Transformer Base backbone, while the remaining RT-DETR detection framework was preserved. The Swin Transformer backbone was selected because its hierarchical multiscale representation and shifted-window self-attention may be useful for hemorrhage subtypes with variable size, morphology, and spatial distribution. RT-DETR was selected because it supports efficient end-to-end object detection using multiscale encoding and Transformer-based object queries. Therefore, Swin-RT-DETR was included as a representative Transformer-based RT-DETR variant for comparative evaluation, rather than as a fundamentally novel detector design. Because Swin-RT-DETR differs from the CNN-based comparators in both backbone design and detection framework, the present comparison was not intended to isolate the independent effect of self-attention mechanisms. Instead, it was designed to compare representative detector architectures under internal and bidirectional external validation settings.

For comparative evaluation, five representative CNN-based object detection architectures were included, covering one-stage and two-stage paradigms: YOLOv8 [26], RetinaNet [27], and Faster R-CNN variants [28]. Detailed architectural configurations are provided in Supplementary S3 (Figures S2 and S3). YOLOv8 and RetinaNet are one-stage detectors optimized for efficient single-pass inference, whereas Faster R-CNN is a two-stage detector based on region proposals.

All models were trained and evaluated under identical hardware conditions using an NVIDIA RTX 3090 GPU (24 GB VRAM) and 32 GB system memory, running Python 3.12.10 and PyTorch 2.5.1. YOLO and Swin-RT-DETR models were implemented using the Ultralytics framework (v8.4.21), while RetinaNet and Faster R-CNN variants were built on MMDetection (mmdet v3.3.0). 

### 2.5. Training Hyperparameters and Run Time Analysis

To ensure reproducibility and improve methodological transparency, the major training hyperparameters for all evaluated models are summarized in Table 1, including optimizer type, learning rate and scheduler, batch size, training epochs, weight decay, loss functions, and data augmentation strategies. Early stopping was applied when specified according to validation performance. Additional model-specific implementation details, architectural configurations, and runtime analyses are provided in the Supplementary S4 (Table S2).

The YOLOv8 variants, including YOLOv8-small and YOLOv8-large, were trained using stochastic gradient descent (SGD) with an initial learning rate of 0.01, momentum of 0.937, and cosine annealing scheduling. Training was performed for up to 80 epochs with early stopping using a patience of 15 epochs. Data augmentation included horizontal flipping, translation with ±10%, scaling, HSV adjustments, RandAugment, random erasing with a probability of 0.4, and mosaic augmentation during the first 70 epochs.

RetinaNet was trained using SGD with an initial learning rate of 0.01, momentum of 0.9, and MultiStepLR scheduling for 50 epochs. Faster R-CNN variants with ResNet101 and ResNeXt101 backbones were trained using SGD with an initial learning rate of 0.001, momentum of 0.9, and MultiStepLR scheduling for 50 epochs. Data augmentation for RetinaNet and Faster R-CNN models was limited to horizontal flipping and resizing.

Swin-RT-DETR was trained using AdamW optimization. Its composite loss function incorporated generalized BB-IoU loss, classification loss, and distribution focal loss to jointly optimize bounding-box regression and subtype classification. Model-specific parameters and runtime performance are shown in Table S3. Training loss curves are provided in Supplementary S5 (Figure S4).

### 2.6. Performance Evaluation

Model performance was evaluated using mAP@50, BB-DSC, and BB-IoU (Equations (S1), (S2) and (S3), respectively, Supplementary S6). Because this study used bounding-box annotations rather than pixel-level masks, DSC and IoU were computed from predicted and ground-truth bounding boxes. These metrics are therefore referred to as BB-DSC and BB-IoU to distinguish bounding-box overlap from pixel-level segmentation accuracy. Precision-recall curves were generated for supplementary performance visualization. The area under the precision–recall curve (AUPRC) was calculated as a supplementary analysis (Equation (S4), Supplementary S6) but was not used for primary performance comparison.

### 2.7. Statistical Analysis

All statistical analyses were performed using SPSS software (version 23) [29]. Image-level and patient-level metrics were summarized for each model and hemorrhage subtype. Data normality was assessed using the Shapiro–Wilk test for sample sizes of 50 or fewer and the Kolmogorov–Smirnov test for larger samples. For comparisons across multiple models evaluated on the same dataset, repeated-measures ANOVA or Friedman tests were used as appropriate according to the distribution of the data. Pairwise comparisons were performed using paired *t*-tests or WRilcoxon signed-rank tests, as appropriate. Bonferroni correction was applied for post hoc pairwise comparisons, with a two-sided p value less than 0.05 considered statistically significant after correction.

Because multiple CT slices may originate from the same patient or scan, image-level statistical comparisons were interpreted as exploratory model-level comparisons. Patient-level analyses were performed as supplementary analyses to reduce slice-level dependence, although they do not fully replace mixed-effects modeling or hierarchical resampling.

## 3. Results

### 3.1. Dataset-Level Differences in Annotation Characteristics

**Figure 4:**
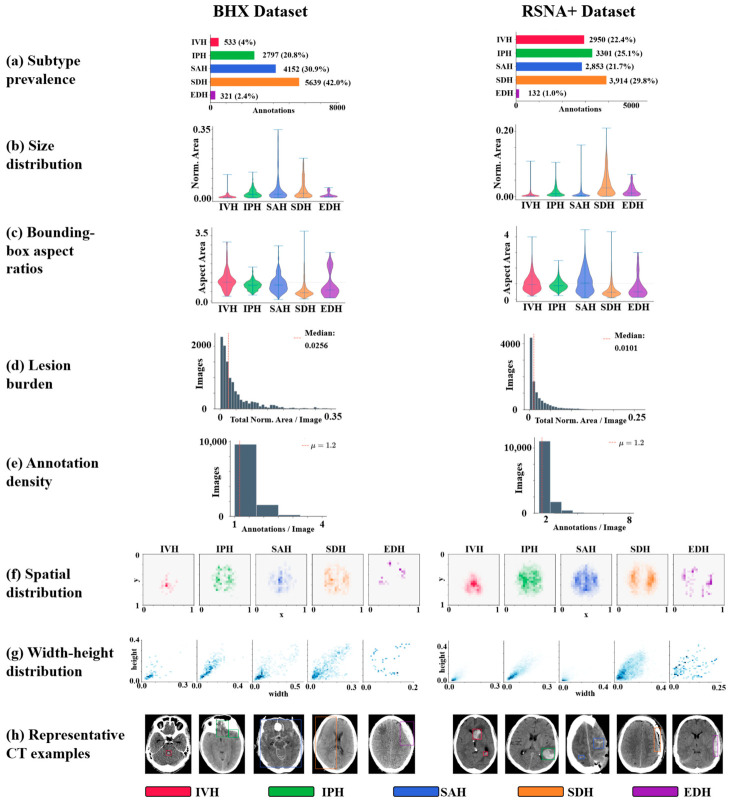
Annotation characteristics of the BHX and RSNA+ datasets. Subtype prevalence (**a**), bounding-box size distribution (**b**), bounding-box aspect ratio (**c**), lesion burden (**d**), annotation density (**e**), spatial distribution (**f**), width–height distribution (**g**), and representative CT examples (**h**) are shown for each intracranial hemorrhage subtype. Dataset-level differences in annotation geometry, lesion burden, subtype distribution, and subtype-specific spatial patterns suggest substantial domain shift between BHX and RSNA+.

To empirically characterize cross-dataset differences, we compared subtype prevalence, bounding-box size, aspect ratio, lesion burden, annotation density, spatial distribution, and representative imaging appearances between the BHX and RSNA+ datasets (Figure 4). Substantial differences were observed between datasets across multiple annotation-related dimensions. The subtype distribution differed between BHX and RSNA+, with variations in the relative prevalence of IVH, IPH, SAH, SDH, and EDH. In addition, normalized bounding-box size and lesion burden distributions showed dataset-specific patterns, suggesting differences in hemorrhage extent and annotation granularity. Bounding-box aspect ratios also varied across subtypes, particularly among extra-axial hemorrhages: SAH frequently showed irregular spatial configurations, whereas SDH more often demonstrated elongated patterns. Annotation density was predominantly one to two annotations per image in both datasets, but the distribution differed between datasets, indicating potential differences in labeling practice or lesion multiplicity.

Spatial distribution analysis further demonstrated subtype-specific and dataset-specific localization patterns. Compact hemorrhage subtypes, such as IPH and IVH, showed relatively clustered distributions. In contrast, SAH demonstrated broader and more irregular spatial variability, whereas SDH showed more elongated width–height profiles consistent with extra-axial crescentic morphology. EDH showed broader variability but fewer annotated cases. These findings provide quantitative evidence that BHX and RSNA+ differed not only in image source but also in annotation geometry, lesion burden, subtype distribution, and localization patterns. Such differences likely contributed to the observed degradation in cross-dataset localization performance.

### 3.2. Localization Performance Under Internal and External Validation

**Figure 5:**
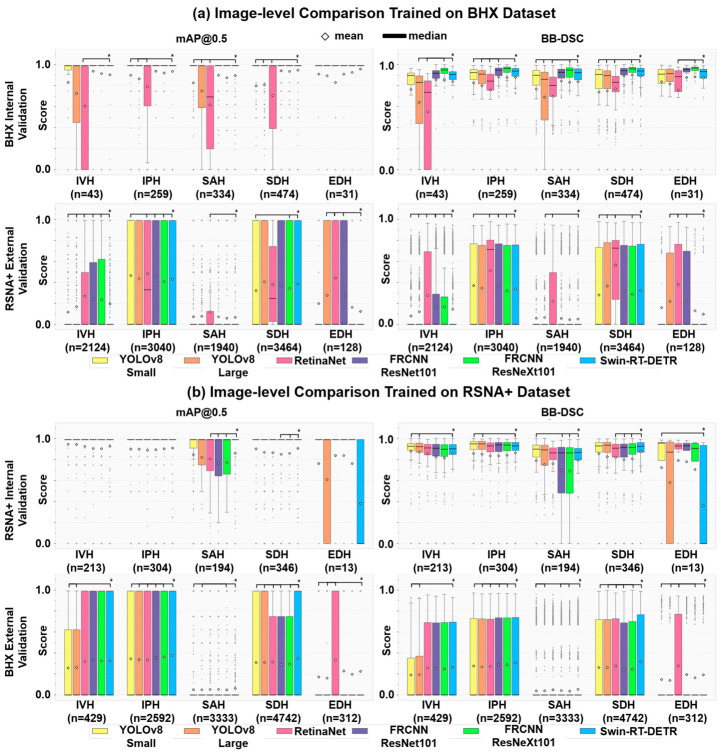
Image-level cross-dataset performance comparison. Models were trained on (**a**) BHX or (**b**) RSNA+ and evaluated using mAP@50 and BB-DSC. Asterisks indicate Bonferroni-corrected significant differences from Swin-RT-DETR.

At the image level, Swin-RT-DETR achieved the highest or among the highest localization performance across most ICH subtypes during internal validation on the BHX dataset (Figure 5a). Swin-RT-DETR attained mAP@50 values of 0.9032, 0.9361, 0.8960, 0.9475, and 0.9582 for IVH, IPH, SAH, SDH, and EDH, respectively. Corresponding BB-DSC values were 0.8344, 0.8751, 0.8385, 0.8909, and 0.8752. These values were significantly higher than those of YOLOv8-small, YOLOv8-large, and RetinaNet for most subtype comparisons after Bonferroni correction. However, the advantage of Swin-RT-DETR was not uniform across all subtypes. Faster R-CNN with a ResNeXt101 backbone showed competitive performance, with comparable IVH BB-DSC and BB-IoU and significantly higher IPH BB-DSC and BB-IoU than Swin-RT-DETR. In contrast, Swin-RT-DETR showed significantly higher BB-DSC and BB-IoU for SAH, SDH, and EDH, indicating subtype-dependent rather than universal superiority. External validation on the RSNA+ test set showed substantial performance degradation across architectures and subtypes (Figure 5a; Supplementary S7, Tables S3 and S4). Swin-RT-DETR achieved BB-DSC values of 0.1440, 0.3368, 0.0504, 0.3228, and 0.0832 for IVH, IPH, SAH, SDH, and EDH, respectively, corresponding to absolute BB-DSC reductions of approximately 0.54–0.79 compared with internal validation. Similar degradation was observed for the competing models. No single architecture consistently maintained the best performance across all subtypes under external validation; for example, RetinaNet achieved the highest BB-DSC for SDH on external validation (0.57) but performed significantly worse than Swin-RT-DETR on internal testing. The morphology-dependent pattern persisted externally, with IPH and SDH maintaining relatively higher BB-DSC values than SAH, IVH, and EDH. The reciprocal experiment, in which models were trained on RSNA+ and evaluated on BHX, showed a similar pattern of limited cross-dataset robustness (Figure 5b; Supplementary S7, Tables S5 and S6). Although internal validation on RSNA+ demonstrated high localization performance for several subtypes, external validation on BHX again resulted in reduced mAP@50 and BB-DSC. This bidirectional degradation indicates that the observed performance loss was not specific to the BHX-to-RSNA+ direction. Instead, the results suggest that dataset composition, annotation geometry, and subtype-specific morphology affected external localization performance in both directions.

**Figure 6:**
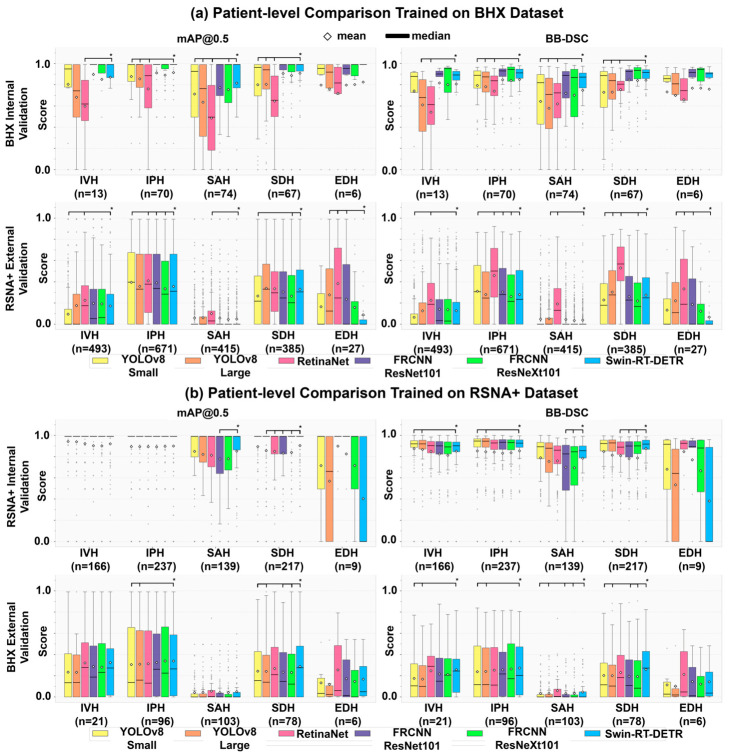
Patient-level cross-dataset performance comparison. Models were trained on (**a**) BHX or (**b**) RSNA+ and evaluated using mAP@50 and BB-DSC. Asterisks indicate Bonferroni-corrected significant differences from Swin-RT-DETR.

Patient-level analysis showed trends broadly consistent with the image-level findings (Figure 6; Supplementary S7, Tables S7–S10). When models were trained on BHX, internal patient-level performance was higher than external patient-level performance on RSNA+. Similarly, when models were trained on RSNA+, patient-level external validation on BHX showed reduced performance compared with internal testing. The patient-level results therefore support the image-level observation that cross-dataset generalization remained limited, although aggregation at the patient level partially reduced the influence of individual slice-level variability.

**Figure 7:**
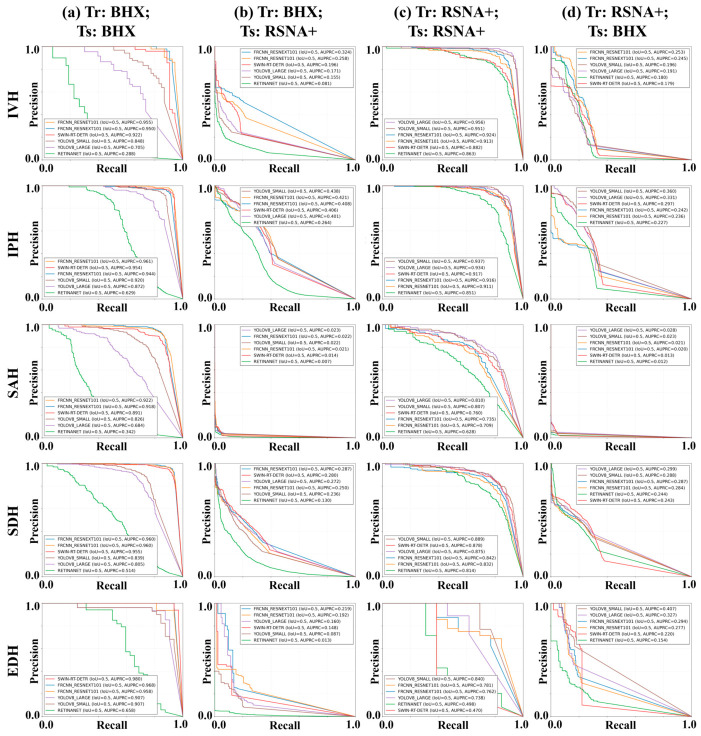
Precision–recall curves under bidirectional cross-dataset validation. Curves and AUPRC values are shown for five ICH subtypes across four training/testing settings: (**a**) trained and tested on BHX, (**b**) trained on BHX and tested on RSNA+, (**c**) trained and tested on RSNA+, and (**d**) trained on RSNA+ and tested on BHX. Tr indicates training dataset; Ts, testing dataset.

Precision–recall curve analysis further supported the cross-dataset performance pattern (Figure 7). In both training directions, internal validation showed higher and more stable precision–recall profiles than external validation. When models were trained on BHX and tested on RSNA+, precision decreased substantially across most recall ranges, particularly for SAH, IVH, and EDH. A similar reduction was observed in the reciprocal RSNA+-to-BHX setting, indicating that degraded external performance was not limited to a single dataset direction. These findings are consistent with the mAP@50 and BB-DSC results and further demonstrate limited cross-dataset robustness across ICH subtypes.

Statistical comparisons further demonstrated that architecture-level differences were less distinct under external validation. Compared with internal validation, external datasets showed fewer statistically significant pairwise differences between models after Bonferroni correction at both the image and patient levels. This attenuation of model-level differences suggests that the relative advantage of individual architectures diminished under cross-dataset conditions. Therefore, although Swin-RT-DETR showed stronger internal performance in several subtype comparisons, the bidirectional external validation results indicate that domain shift and annotation heterogeneity may exert a greater influence on localization performance than architecture-specific differences alone.

Across architectures, compact hemorrhage patterns, such as IPH, showed higher and more stable localization performance, whereas morphologically complex subtypes, particularly irregular or diffuse SAH and elongated extra-axial SDH, showed lower and more variable performance, suggesting a morphology-dependent performance pattern (Figure 5a,b).

### 3.3. Qualitative Interpretability Analysis

**Figure 8:**
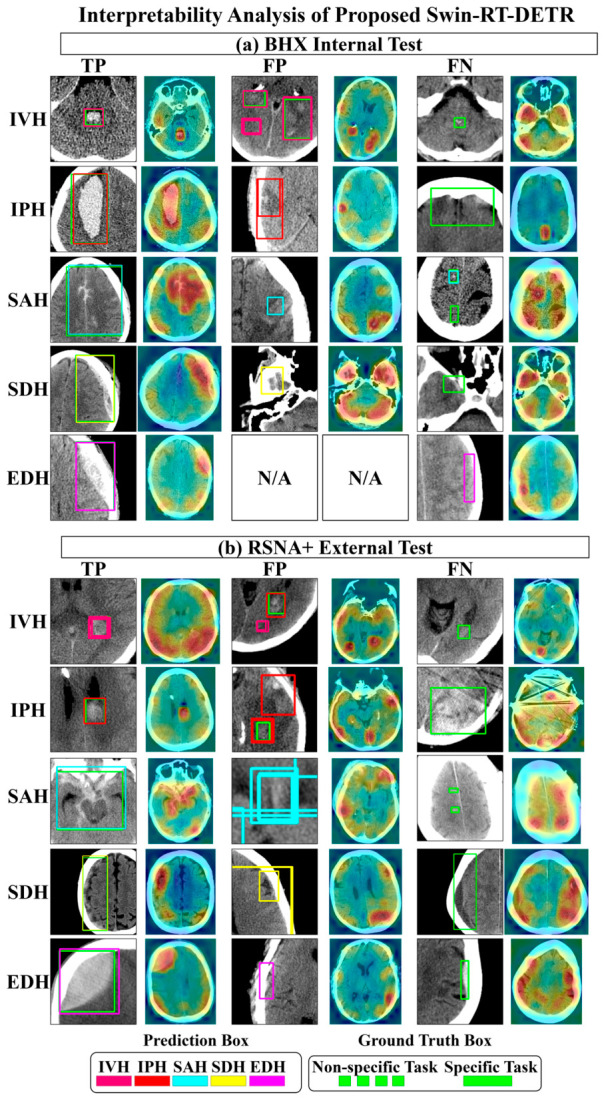
Interpretability analysis of Swin-RT-DETR on BHX internal and RSNA+ external test sets. Representative true-positive, false-positive, and false-negative cases are shown for each ICH subtype. Attention maps illustrate subtype-specific activation patterns and failure modes under internal and external validation. N/A denotes not available. The proposed Swin-RT-DETR did not have any false positive predictions for EDH.

Interpretability analysis of Swin-RT-DETR demonstrated that true-positive localizations generally showed attention concentrated near hemorrhage-related regions on both the BHX internal test set and the RSNA+ external test set (Figure 8). In contrast, false-positive cases frequently showed activation in anatomically plausible but non-hemorrhagic regions, such as sulcal hyperdensity, skull-adjacent structures, or extra-axial spaces. False-negative cases often involved small, subtle, diffuse, or low-contrast hemorrhages, particularly for SAH and EDH. These findings suggest that model failure under external validation was not only related to localization thresholding but also to subtype-specific morphology and dataset-dependent imaging or annotation characteristics.

## 4. Discussion

In this study, we systematically evaluated object localization frameworks for ICH subtype localization on noncontrast head CT, with particular emphasis on the effect of detector architecture and cross-dataset generalizability. The principal finding was that Transformer-based hierarchical feature extraction improved internal localization performance in several subtypes; however, this advantage was substantially attenuated under external validation. These results suggest that dataset and annotation shifts may have a greater influence on external localization performance than architecture-specific differences alone.

The dataset characterization analysis further clarified that the external performance degradation was not solely attributable to model architecture. BHX and RSNA+ differed in subtype prevalence, bounding-box size, aspect ratio, lesion burden, annotation density, and spatial distribution. These discrepancies indicate that cross-dataset evaluation involved both imaging-domain shift and annotation-domain shift. In particular, differences in bounding-box geometry may disproportionately affect BB-DSC and BB-IoU because these metrics quantify rectangular box overlap rather than pixel-level lesion conformity. This issue is especially relevant for SAH and SDH, but for different geometric reasons: irregular, diffuse, or multifocal SAH and elongated, crescentic SDH are both poorly represented by a single rectangular bounding box. Therefore, the reduced external performance should be interpreted as evidence of limited cross-dataset robustness under heterogeneous annotation and morphology conditions, rather than as failure of one specific architecture alone.

Previous ICH localization studies have predominantly focused on CNN-based detectors evaluated within a single dataset, most commonly [9,10,11,12,13,22]. Ertugrul et al. and Kothala et al. reported improved recall and F1 scores using separated or single-class training strategies [9,13]; however, these conclusions were derived exclusively from internal train–test splits without external validation. Similarly, Ferdi et al. demonstrated improved internal performance using an enhanced YOLOv8 architecture [11], but cross-dataset generalization was not assessed. More recent studies incorporating external validation have reported substantial performance degradation, particularly for EDH. Tapia et al. observed EDH performance collapse on an external Chilean dataset despite strong internal results [15], while Liu et al. reported poor EDH generalization when training on RSNA+ and testing on CQ500+, attributing this primarily to class imbalance and limited EDH samples [14].

Our findings are consistent with these observations. At the image level, Swin-RT-DETR achieved competitive or superior internal validation performance across several ICH subtypes when trained and tested on BHX. However, this advantage was subtype-dependent rather than uniform. Faster R-CNN with a ResNeXt101 backbone achieved comparable IVH BB-DSC and BB-IoU and significantly higher IPH BB-DSC and BB-IoU, whereas Swin-RT-DETR showed clearer advantages for SAH, SDH, and EDH. This finding indicates that Transformer-based hierarchical feature extraction may benefit selected morphologically complex subtypes, but it does not confer universal superiority across all hemorrhage categories.

More importantly, external validation demonstrated substantial performance degradation across architectures, subtypes, and validation directions. When models were trained on BHX and externally tested on RSNA+, image-level BB-DSC decreased markedly across all subtypes. The reciprocal experiment, in which models were trained on RSNA+ and externally tested on BHX, showed a similar reduction in external performance. Patient-level analysis demonstrated broadly consistent trends, with higher internal performance than external performance in both training directions. Although patient-level aggregation may reduce the influence of individual slice-level variability, it did not eliminate cross-dataset performance degradation. These bidirectional image-level and patient-level findings suggest that the observed performance loss was not a unidirectional artifact of BHX-specific optimization or RSNA+-specific testing conditions.

The statistical findings should therefore be interpreted not only according to which model achieved the highest metric values, but also according to how the pattern of significance changed between validation settings. Under internal validation, several significant pairwise differences suggested architecture-related performance advantages. Under external validation, however, fewer model-level differences remained statistically significant, and the relative ranking of architectures varied across subtypes and validation directions. This attenuation of statistical separation supports the interpretation that dataset shift, annotation heterogeneity, and subtype-specific morphology reduced the practical importance of architecture-specific gains.

Swin-RT-DETR was included to assess the potential value of Transformer-based hierarchical feature extraction for ICH localization rather than to propose a fundamentally new localization architecture. The Swin Transformer backbone was selected because its hierarchical representation and shifted-window self-attention mechanism provide multiscale contextual feature extraction, which may be relevant for hemorrhage subtypes with heterogeneous size, morphology, and spatial distribution. RT-DETR was used as the localization framework because it supports efficient end-to-end object localization through multiscale encoding and Transformer-based object queries. These properties may partly explain the stronger internal localization performance of Swin-RT-DETR compared with several CNN-based baselines. Nevertheless, the external validation results showed that these architecture-related advantages were substantially reduced under cross-dataset conditions, indicating that architectural design alone is insufficient to overcome dataset and annotation shifts.

Nevertheless, the present comparison cannot isolate the independent contribution of self-attention from other architectural factors, because Swin-RT-DETR differs from the CNN-based comparators in both backbone design and detection framework. Therefore, the observed internal performance advantage should be interpreted as the performance of one representative Transformer-based RT-DETR variant rather than as direct evidence that attention mechanisms alone improve ICH localization.

Across architectures and datasets, localization performance consistently depended on hemorrhage morphology. Compact subtypes such as IPH and IVH were localized with higher accuracy [14], whereas spatially diffuse or thin extra-axial subtypes, particularly SAH and SDH, showed lower and more variable performance [10,14]. This morphology-dependent hierarchy persisted under both internal and cross-dataset validation, suggesting that it may reflect intrinsic geometric constraints of bounding box–based localization rather than model-specific deficiencies alone. Compact hemorrhages occupy well-defined spatial regions that align naturally with rectangular bounding boxes, facilitating accurate regression. In contrast, elongated crescentic SDH often yields boxes with high aspect ratios and substantial background inclusion, whereas irregular or multifocal SAH may require boxes that encompass noncontiguous hemorrhagic regions and intervening normal tissue, reducing BB-IoU and BB-DSC even when localization is visually plausible. Irregular or diffuse SAH distributed across multiple noncontiguous sulci poses an additional challenge because no single bounding box can tightly enclose the hemorrhagic regions without also encompassing intervening normal tissue. Throughout this study, localization refers to bounding-box localization rather than pixel-level lesion segmentation. Therefore, BB-DSC and BB-IoU should be interpreted as measures of rectangular box overlap, not as indicators of anatomical lesion-contour accuracy. This distinction is particularly relevant for SAH and SDH: irregular, diffuse, or multifocal SAH and elongated, crescentic SDH are poorly represented by a single rectangular bounding box and may include substantial normal tissue. These morphological constraints likely represent an inherent limitation of object detection–based frameworks and suggest that future work may benefit from segmentation or hybrid detection–segmentation approaches for subtypes with irregular spatial distributions.

The clinical implications of these findings should be interpreted cautiously and according to the intended use case. No universally accepted minimum BB-DSC, BB-IoU, or mAP@50 threshold currently defines clinical readiness for ICH localization models. A triage-support system may prioritize high sensitivity and an acceptable false-positive burden, whereas visual prompting for radiologist review may tolerate less precise localization if suspicious regions are reliably highlighted. In contrast, lesion follow-up, volumetric assessment, or treatment-response monitoring would require substantially more accurate spatial delineation, for which segmentation-based approaches may be more appropriate.

In the present study, the low external BB-DSC values for several subtypes, particularly IVH, SAH, and EDH, indicate that the evaluated models are not yet suitable for stand-alone clinical deployment. At the current level of external performance, their potential role should be considered investigational and adjunctive rather than autonomous. Possible use cases may include radiologist triage support, visual prompting of suspected hemorrhage regions, quality-control review, or assistance in research annotation workflows. However, these applications require prospective validation to determine whether automated localization improves reader sensitivity, reduces interpretation time, or affects clinical decision-making without increasing false-positive burden. Therefore, the present study should be interpreted as demonstrating the limitations and fragility of current cross-dataset ICH localization systems rather than establishing readiness for clinical deployment.

Several limitations warrant consideration. First, although our multi-dataset design provides insight into cross-dataset generalization, validation was limited to two publicly available datasets. Further evaluation across additional institutions, scanner manufacturers, acquisition protocols, and patient populations is needed to confirm generalizability. In addition, although we added dataset characterization analyses to examine annotation-related sources of domain shift, scanner parameters, acquisition protocols, and population-level variables were not consistently available across the public datasets. Therefore, the relative contributions of scanner-related differences, population bias, and annotation-domain shift could not be fully disentangled. Second, this study focused on 2D slice-level bounding-box localization, which does not fully capture volumetric hemorrhage extent or patient-level diagnostic decision-making. Several primary statistical comparisons were based on image-level metrics. Because multiple CT slices may originate from the same patient or scan, slice-level observations may not be fully independent, which could inflate statistical significance. Although patient-level analyses were added and showed trends broadly consistent with the image-level results, patient-level aggregation alone does not fully model within-patient correlation or uncertainty. Therefore, Bonferroni-corrected p values should be interpreted as exploratory model-level comparisons rather than definitive patient-level inference. Future studies should incorporate scan-level bootstrapping, hierarchical resampling, generalized estimating equations, or mixed-effects modeling to provide more robust estimates of model performance and statistical uncertainty. Third, the analysis was restricted to single-class labeled images to reduce label ambiguity and allow controlled subtype-specific comparisons across datasets and architectures. However, this design simplifies the clinical task because hemorrhage subtypes frequently coexist, and multi-class images may contain useful anatomical context for differentiating hemorrhage patterns. Therefore, the reported performance may not fully represent real-world ICH localization complexity. Future studies should evaluate multi-label or multi-class detection frameworks that allow simultaneous localization of coexisting hemorrhage subtypes. Fourth, annotation variability between the BHX and RSNA+ datasets, including differences in annotator expertise, labeling conventions, and bounding-box granularity, may have contributed to the observed domain shift beyond imaging-related differences. Fifth, the relatively small number of EDH instances in both datasets limits the statistical power of subtype-specific comparisons for this category. Sixth, although Swin-RT-DETR was included as a representative Transformer-based RT-DETR variant, this study did not exhaustively compare all available Transformer-based detectors. Alternative architectures, including deformable DETR variants, DINO-based detectors, and other vision Transformer backbones, may provide different trade-offs in convergence behavior, small-object sensitivity, computational cost, and cross-dataset robustness. In addition, the current model comparison cannot fully isolate the contribution of self-attention mechanisms from broader architectural differences, because Swin-RT-DETR differs from the CNN-based detectors in both backbone and detection framework. Future studies should include pure Transformer detectors, additional modern CNN detectors, and controlled backbone-ablation experiments to disentangle the effects of attention mechanisms, multiscale feature extraction, and detector design. Finally, the clinical impact of automated localization on reader performance, workflow efficiency, false-positive burden, and patient outcomes was not evaluated; prospective studies are needed to establish clinical utility.

## 5. Conclusions

In summary, ICH subtype localization on noncontrast head CT is influenced by both detector architecture and dataset-related generalization factors. Although Swin-RT-DETR showed subtype-dependent advantages under internal validation, its performance decreased substantially under external validation, indicating limited cross-dataset robustness. Dataset composition, annotation geometry, lesion burden, annotation heterogeneity, and hemorrhage morphology substantially affected localization performance, particularly for thin, diffuse, or rare subtypes such as SAH and EDH. The low external localization performance for several subtypes indicates that the evaluated models are not yet suitable for stand-alone clinical deployment. These findings emphasize that external validation, dataset characterization, patient-level evaluation, and task-specific clinical benchmarks are essential before automated ICH localization models can be considered for real-world clinical integration.

## Figures and Tables

**Table 1:** Summary of Training Hyperparameters for All Models.

Model	Optimizer	LR and Scheduler	Batch Size	Epochs	Weight Decay	Loss Function(s)	Data Augmentation
YOLOv8-small	SGD	LR = 0.01, Momentum = 0.937; Cosine annealing	4	80; early stop patience = 15	5 × 10^−4^	Bounding-box loss, classification loss, and distribution focal loss	Flip (50%); translation, ±10%; scale, 50%; HSV; RandAugment; random erasing, *p* = 0.4; mosaic augmentation during the first 70 epochs
YOLOv8-large	SGD	LR = 0.01, Momentum = 0.937; Cosine annealing	4	80; early stop patience = 15	5 × 10^−4^	Bounding-box loss, classification loss, and distribution focal loss	Same as YOLOv8-small
RetinaNet	SGD	LR = 0.01, Momentum = 0.9; MultiStepLR	2	50	1 × 10^−4^	L1 loss and focal loss	Horizontal flip, 50%; resizing
FRCNN (ResNet101)	SGD	LR = 0.001, Momentum = 0.9; MultiStepLR	2	50	5 × 10^−4^	L1 loss and cross-entropy loss	Horizontal flip, 50%; resizing
FRCNN (ResNeXt101)	SGD	LR = 0.001, Momentum = 0.9; MultiStepLR	2	50	5 × 10^−4^	L1 loss and cross-entropy loss	Horizontal flip, 50%; resizing
Swin-RT-DETR	AdamW	LR = 0.0001, betas = (0.9, 0.999)	8	80; early stop patience = 15	5 × 10^−4^	Generalized BB-IoU loss, classification loss, and distribution focal loss	Same as YOLOv8-small

Note: SGD indicates stochastic gradient descent; LR, learning rate; AdamW, Adam with decoupled weight decay; BB-IoU, bounding-box intersection over union; and MultiStepLR, multistep learning-rate scheduler. Early stopping was applied according to validation performance when specified.

## Data Availability

The datasets used in this study are publicly available. The Brain Hemorrhage Extended (BHX) dataset is available at PhysioNet (https://physionet.org/content/bhx-brain-bounding-box/1.1/) (accessed on 1 November 2025). The RSNA 2019+ dataset is available at (http://www.multi-lesions.top) as provided by Liu et al [14] (accessed on 25 November 2025). Additional data generated or analyzed during this study are available from the corresponding author upon reasonable request.

## Supplementary Materials

The following supporting information can be downloaded at: https://www.mdpi.com/article/10.3390/diagnostics16111705/s1, Figure S1: Generation of a multi-window three-channel image from individual CT slices; Figure S2: Schematic illustration of the YOLOv8 architecture; Figure S3: Architectures of the object localization models evaluated in this study; Figure S4: Training Curves on BHX Training Dataset and RSNA+ Dataset; Table S1: Demographic Characteristics and Slice Thickness Across Datasets; Table S2. Comparison of Model Parameters and Runtime Performance across Different Architectures; Tables S3–S6: Image-level quantitative results across all cross-dataset scenarios; Tables S7–S10: Patient-level quantitative results across all cross-dataset scenarios. [14,22,25,26,27,28,30,31].

## Abbreviations

The following abbreviations are used in this manuscript:

CNN	Convolutional Neural Network
ICH	Intracranial Hemorrhage
BB-DSC	Bounding-box Dice Similarity Coefficient
BB-IoU	Bounding-box Intersection over Union
mAP@50	Mean Average Precision at a bounding-box intersection of union threshold of 0.5
IPH	Intraparenchymal Hemorrhage
IVH	Intraventricular Hemorrhage
SAH	Subarachnoid Hemorrhage
SDH	Subdural Hemorrhage
EDH	Epidural Hemorrhage
