# Supplementary material for: Cross-Dataset Generalization of Deep Learning-Based Detectors for Intracranial Hemorrhage Subtype Localization on Noncontrast Head CT: A Comparative Study
Source: Diagnostics (Basel). 2026 Jun 2;16(11):1705. doi: 10.3390/diagnostics16111705 (PMC13256447; doi:10.3390/diagnostics16111705)
Supplement: Supplementary file 1 [file diagnostics-16-01705-s001.zip › diagnostics-4305002-supplementary.pdf]

Supplementary Materials

Supplementary S1. Dataset Characteristics and Annotation Protocols

Dataset characteristics, including the number of patients, scans, and image slices, as well as demographic information (sex and age), and acquisition parameters (slice thickness), for the RSNA 2019 and the CQ500 datasets are summarized in Table S1. The RSNA 2019 dataset constitutes the source dataset of the RSNA 2019+, whereas the CQ500 dataset serves as the origin of both the BHX dataset and CQ500+ datasets. The RSNA 2019+ dataset [1] consists of non-contrast head CT scans collected from three international medical centers and was publicly released in 2019 with predefined training and testing splits [2]. The training cohort comprised 18,938 patients with 21,744 scans (752,803 slices), while the testing cohort included 3,518 patients with 3,518 scans (121,232 slices). For both cohorts, slice thickness ranged from 3 to 5 mm. The BHX dataset [3] is an annotated subset of the CQ500 dataset that provides structured bounding-box annotations exclusively for intracranial hemorrhage (ICH)-positive cases. It includes five acute hemorrhage subtypes – epidural hemorrhage (EDH), intraparenchymal hemorrhage (IPH), intraventricular hemorrhage (IVH), subarachnoid hemorrhage (SAH), and subdural hemorrhage (SDH) – as well as chronic subdural hematoma. To address heterogeneity in imaging acquisition and annotation granularity, three annotation variants are provided: manually annotated thick-slice images, fully extrapolated annotations, and a curated soft-tissue thin-slice subset comprising 15,979 images with 27,203 bounding boxes. The CQ500+ dataset [1] includes 491 non-contrast head CT scans (261 ICH-negative and 230 ICH-positive) acquired from six radiology centers in New Delhi, India between January 2012 and February 2018. The cohort consisted of 313 males (63.7%) and 178 females (36.3%), with a mean age of 48.1 years (range 7–95 years) (Fig. S1). Slice thickness ranged from 0.625 to 0.5 mm. All data were anonymized in compliance with HIPAA-regulations. Inclusion criteria required the availability of non-contrast CT images, patient age at least 7 years, and corresponding radiology reports. Exclusion criteria included postoperative scans, missing non-contrast CT data, and patients younger than 7 years).

For the BHX dataset [3], six distinct labels were created: five corresponding to acute hemorrhage subtypes—intraparenchymal, subarachnoid, intraventricular, epidural, and subdural hemorrhage—and a sixth label for chronic subdural hematoma. Annotation of the thick-slice series was performed by three neuroradiologists with varying clinical experience: one with six years (F.N.), one with four years (M.A.), and one with less than one year (E.R.). All head CT images were evaluated using a soft tissue filter. Thick-slice series, defined as those with a slice thickness of 3 mm or greater, and thin-slice series, defined as those with a slice thickness of 1 mm or less, were selected and matched using the "Image Position (patient)" DICOM tag. In the creation of the RSNA 2019+ dataset [1], the specialized tool labelImg was employed to annotate the bounding box and category for each intracranial hemorrhage (ICH) in the CT slices, using the category labels "IVH," "IPH," "SAH," "SDH," and "EDH." The annotation process followed consistent principles: each ICH was annotated according to its smallest external rectangle, and different ICH subtypes were annotated separately, even if spatially connected. The annotations were performed jointly by two radiologists with over 13 years of pretraining experience. In cases where a hemorrhage was difficult to annotate, they discussed it with each other, and any disagreements were resolved by a third radiologist with over 20 years of experience, who made the final decision.

Table S1. Demographic Characteristics and Slice Thickness Across.

Characteristics	RSNA 2019 Testing Set	CQ500
**Patients, n**	3,518	491
**Scans, n**	3,518	491
**Slices, n**	121,232	21,418
**Male, n (%)**	N/A	313 (63.7%)
**Female, n (%)**	N/A	178 (36.3%)
**Age (years), mean (range)**	N/A	48.1 (7-95)
**Slice thickness (mm)**	3-5	0.625-5

Note; n denotes number; N/A denotes not available.

Supplementary S2. Image Preprocessing

Imaging preprocessing is illustrated in Fig. S1. All non-contrast CT images were processed using three standardized window settings: brain window (window width [WW] = 80, window level [WL] = 40), subdural window (WW = 50, WL = 175), and bone window (WW = 3000, WL = 500) to incorporate complementary tissue contrast information. Background removal was then applied independently to each windowed image using the following steps: (1) fixed thresholding (> 10 HU) to remove background air and non-relevant regions, (2) morphological opening and closing to refine the foreground mask, (3) extraction of the largest connected component to isolate the head region, and (4) element-wise multiplication to remove pixels outside the head mask. Finally, the three windowed images were concatenated to form a three-channel image, enabling the model to leverage multi-window information, consistent with a previously reported approach [1].

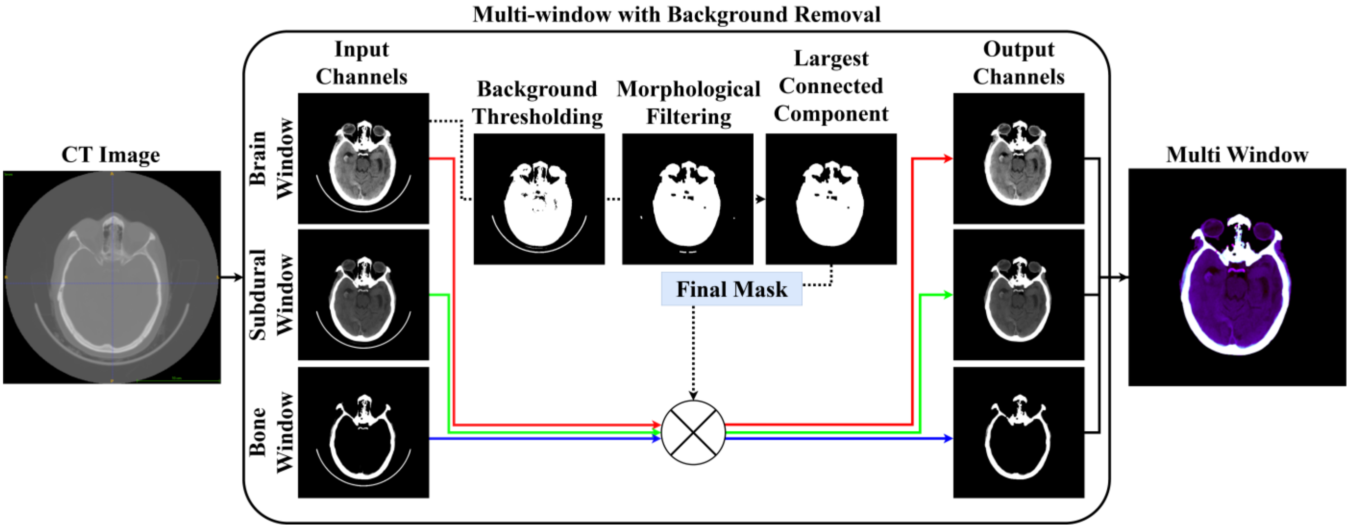

Figure S1. Generation of a multi-window three-channel image from individual CT slices through background removal, followed by integration of three standardized window settings.

Supplementary S3. Detailed Deep Learning Models

Fig. S2 illustrates the baseline YOLOv8 architecture, which employs a hierarchical feature extraction design through three primary pyramid levels (P3, P4, and P5). Multi-scale feature aggregation is achieved using Spatial Pyramid Pooling-Fast and Cross-Stage Partial modules with shortcut connections, enabling efficient representation learning while maintaining computational efficiency [4] (Fig. S2). The detection head employs a decoupled architecture separating classification and localization tasks, with bounding box regression loss serving as the primary optimization objective.

Fig. S3 illustrates the architectures of two object detection models evaluated in this study. The RetinaNet architecture is built on a ResNet-50 backbone with a Feature Pyramid Network, extracting multi-scale features from pyramid levels P3 to P7, which are

subsequently processed by parallel classification and bounding box regression subnets to enable scale-invariant detection (Fig. S3a). To addresses class imbalance, RetinaNet incorporates focal loss, which down-weights well-classified samples and emphasizes hard examples during training [5]. The Faster R-CNN (FRCNN) [6] variants adopt a two-stage detection framework consisting of a Region Proposal Network followed by region-of-interest-based classification and localization refinement (Fig. S3b). Multiple backbone architectures were evaluated, including ResNet-101 [7] and ResNeXt-101 [8]. These backbones represent increasing model complexity and representational capacity, enabling systematic evaluation of the relationship between backbone expressiveness and hemorrhage localization performance.

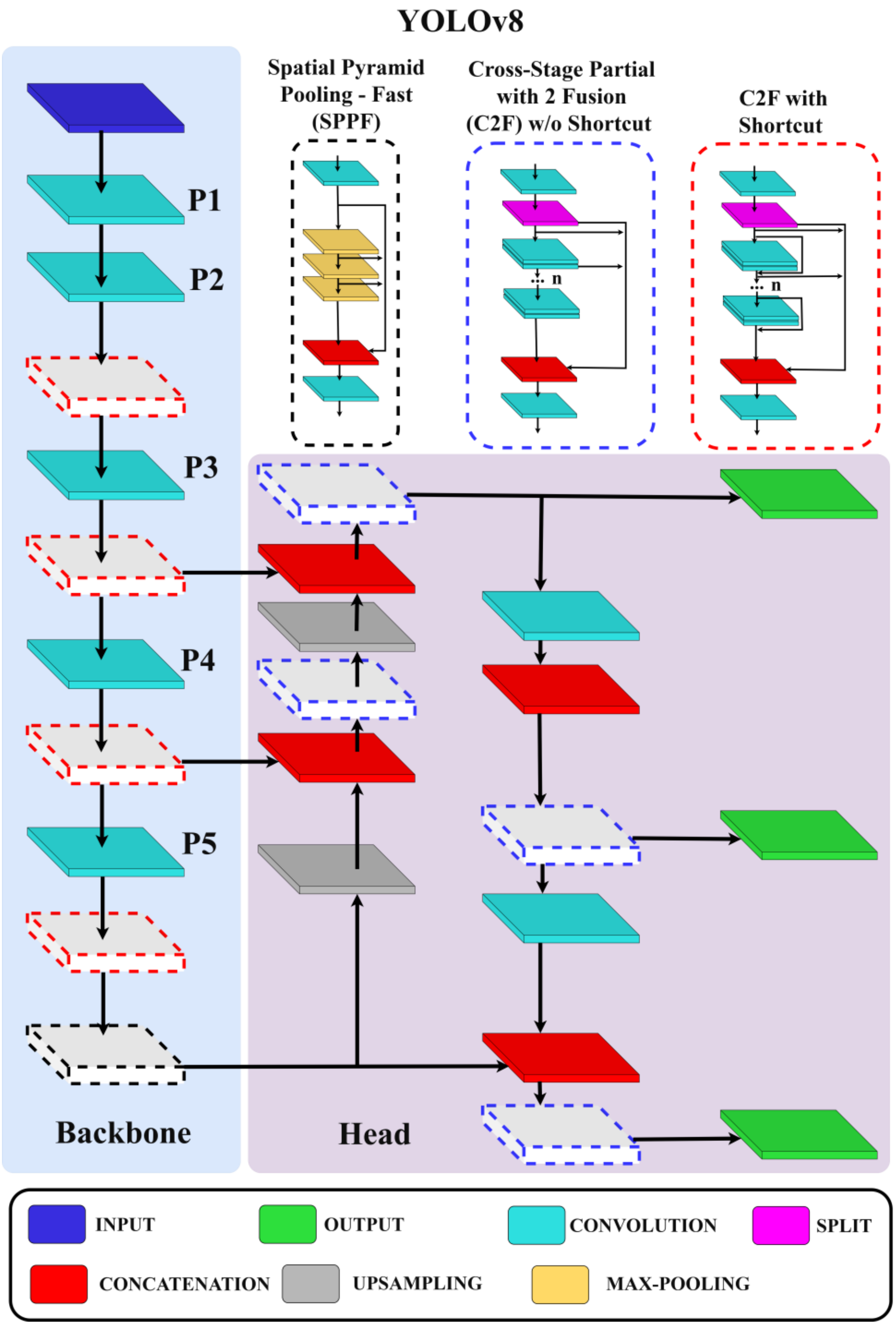

Figure S2. Schematic illustration of the YOLOv8 architecture, including the backbone with multi-scale feature pyramid levels (P3–P5), Spatial Pyramid Pooling–Fast (SPPF), Cross-Stage Partial

(C2F) modules with shortcut connections, and a decoupled detection head for classification and bounding box regression.

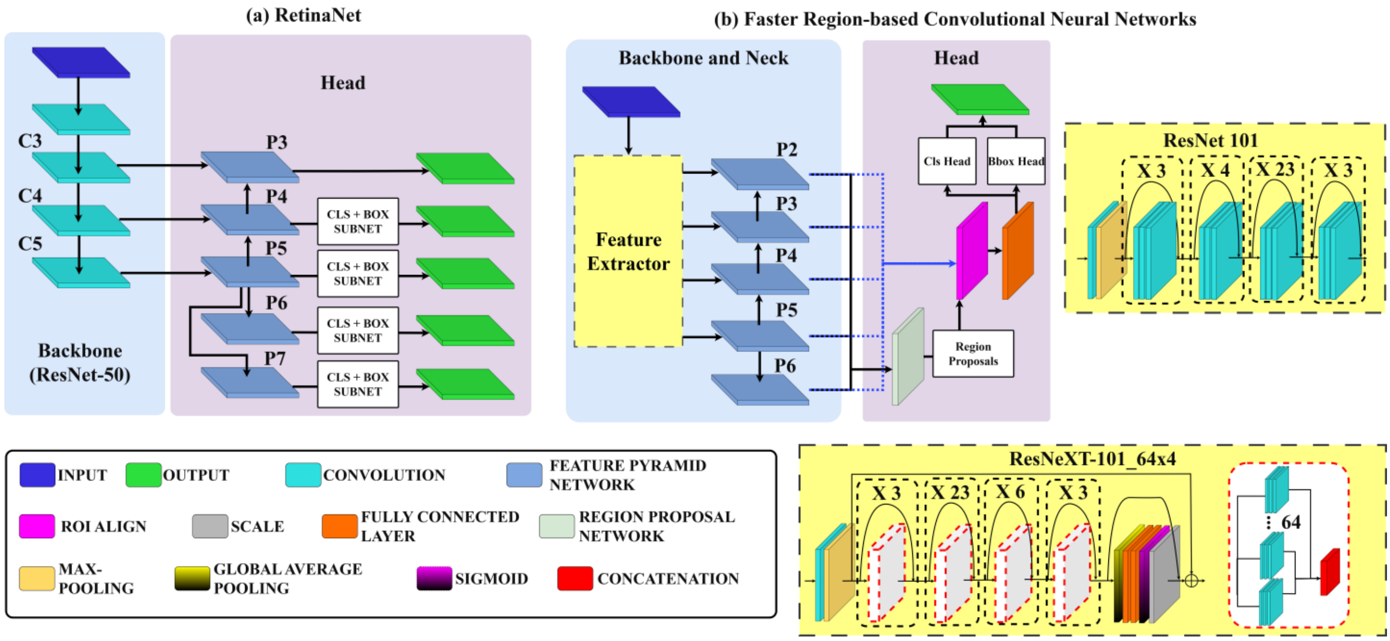

Figure S3. Architectures of the object detection models evaluated in this study: (a) RetinaNet with a Feature Pyramid Network backbone and (b) Faster R-CNN variants employing a two-stage detection framework with multiple backbone architectures including ResNet 101 and ResNeXT-101.

Supplementary S4. Training Hyperparameters and Run Time Analysis

Table S2 provides a comparison of computational efficiency and resource requirements across the six evaluated architectures. The analysis reveals a clear trade-off between model complexity and computational demands, with YOLOv8 small being the most efficient model featuring only 2.7 million parameters, the smallest model size (0.022 GB), and the fastest inference time (0.036 s/image). In contrast, FRCNN with ResNet101 and RetinaNet, both containing 60 and 36 million parameters, respectively, demonstrate significantly higher computational overhead with model sizes of 0.508 GB and 0.313 GB respectively, and substantially longer training times (4.430 and 2.802 s/image). However, despite the extensive training time requirements, the inference times for FRCNN (0.082 s/image) and RetinaNet (0.086 s/image) remain relatively comparable to the YOLO variants, suggesting that the computational burden is primarily concentrated during the training phase rather than deployment. This observation, combined with FRCNN's superior detection performance demonstrated in Table S2, indicates significant potential for optimizing this architecture to achieve both high precision and stable performance in clinical applications where accuracy is essential

Table S2. Comparison of Model Parameters and Runtime Performance across Different Architectures.

Method	Parameters (M)	Model Size (GB)	Training Image (n)	Training Time (s)	Training Throughput (image/s)	Testing Image (n)	Inference Time (s)	Inference Throughput (image/s)
YOLOv8-small	2.70	0.022		3,778	2.72		41	27.83
YOLOv8-large	57.4	0.0860		19,090	0.54		58	19.67
RetinaNet	36.41	0.3130	10,267	28,772	0.36	1,141	98	11.64
FRCNN (resnet101)	60.36	0.5080		45,486	0.23		94	12.14
FRCNN (resnext101)	99.27	0.8117		71,468	0.14		111	10.28

Swin-RT-DETR	102.3	0.4053	55,200	0.19	109.5	10.42

Note: M denotes million parameters; GB denotes gigabytes; images/s denotes images per second; FRCNN denotes faster R-CNN. Training and inference times were measured on the same hardware configuration described in the Methods section.

Supplementary S5. Detailed Training Curves

Fig. S4 presents comprehensive training dynamics across six deep learning architectures evaluated on both the BHX and RSNA+ training datasets, revealing distinct convergence patterns and optimization characteristics. On the BHX dataset (Fig. S4a), the YOLOv8 variants demonstrate rapid initial convergence within the first ten epochs, with training loss, classification loss, and bounding box regression loss stabilizing after approximately twenty epochs across both model sizes (small and large). This rapid convergence behavior is characteristic of YOLO architectures, which are designed to process images in real-time at high frame rates while maintaining detection accuracy. The consistent convergence patterns across different YOLOv8 scales suggest robust optimization dynamics inherent to the architecture, though the larger variant exhibits slightly smoother loss curves, indicating enhanced representational capacity. The FRCNN variants demonstrate progressively smoother convergence characteristics correlating with backbone complexity. The RetinaNet architecture displays distinct training behavior characterized by a pronounced initial loss reduction followed by extended plateau phases, with the focal loss component exhibiting more gradual optimization compared to bounding box regression loss. The Swin-RT-DETR architecture achieved the most stable optimization trajectory and lowest final loss values. Training dynamics on the RSNA+ dataset (Fig. S4b) followed broadly similar convergence patterns across all architectures, although slightly different loss magnitudes were observed, consistent with differences in dataset size and annotation characteristics between the two datasets.

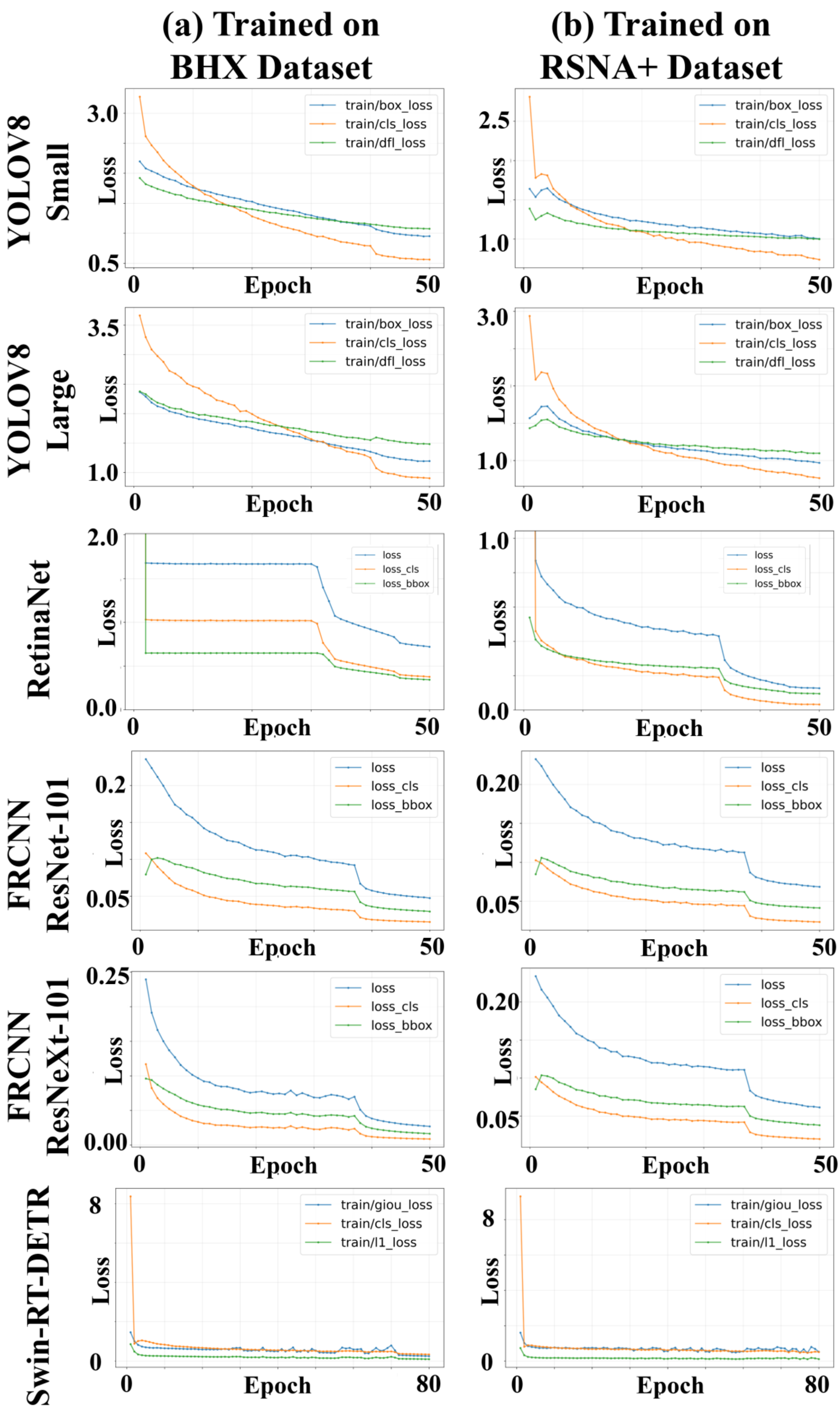
(a) Trained on BHX Dataset
(b) Trained on RSNA+ Dataset
YOLOV8 Small
YOLOV8 Large
RetinaNet
FRCNN ResNet-101
FRCNN ResNeXt-101
Swin-RT-DETR
Loss
Epoch
train/box_loss
train/cls_loss
train/dfl_loss
loss
loss_cls
loss_bbox
train/giou_loss
train/l1_loss

Figure S4. Training Curves of YOLOv8 Small, YOLOv8 Large, RetinaNet, FRCNN ResNet101, FRCNN ResNeXt101, and Swin-RT-DETR Trained on (a) BHX Dataset and (b) RSNA+ Dataset.

Supplementary S6. Performance Evaluation

Mean average precision at an intersection over union threshold of 0.5 (mAP@50) measures the mean average detection precision across all classes when predictions are considered correct if their IoU with ground truth is at least 0.5, reflecting moderate localization accuracy (Eq. S1). The Bounding Box-Dice similarity coefficient (BB-DSC) evaluates spatial overlap by jointly accounting for false-positive and false-negative detections and is commonly used to assess overlap consistency between predicted box and reference regions box (Eq. S2). Bounding Box-Intersection over union (BB-IoU) quantifies the ratio of the overlapping box areas to the union of the predicted and ground truth regions, providing a complementary measure of localization accuracy (Eq. S3).

The area under the precision-recall curve (AUPRC) summarizes the trade-off between precision and recall across all confidence thresholds and is particularly informative for imbalanced datasets, such as those encountered in hemorrhage detection tasks (Eq. S4). All evaluation metrics were computed on a per-class basis and averaged across classes.

Evaluation metrics:

$$mAP@50 = \frac{1}{C}\sum_{c=1}^{C}\int_0^1 P_c\,(R)dR \quad at\ IoU\ \geq 0.5 \quad \text{(S1)}$$

$$BB - DSC = \frac{2 \times TP}{2\times TP + (FP + FN)} \quad \text{(S2)}$$

$$BB - IoU = \frac{TP}{(TP + FP + FN)} \quad \text{(S3)}$$

$$AUPRC = \int_0^1 P(R)dR \quad \text{(S4)}$$

where TP, TN, FP, and FN denote true positive, true negative, false positive, and false negative; C is the number hemorrhage subtypes; and P(R) denotes precision as a function of recall.

Supplementary S7. Detailed Quantitative Results

Tables S3-S10 provide comprehensive comparisons of quantitative results of all cross-dataset scenarios with 95% confidence interval.

Table S3. Image-level Quantitative Results of Deep Learning Models Trained on BHX Dataset and Tested on BHX Dataset.

Model	ICH Subtypes	mAP@50			BB-DSC			BB-IoU		
		Mean ± Std	95% Confidence Interval		Mean ± Std	95% Confidence Interval		Mean ± Std	95% Confidence Interval	
			Lower Bound	Upper Bound		Lower Bound	Upper Bound		Lower Bound	Upper Bound
YOLOV8 small	IVH	0.83 ± 0.33	0.73	0.93	0.76 ± 0.30	0.67	0.86	0.69 ± 0.28	0.61	0.78
	IPH	0.90 ± 0.27	0.87	0.93	0.83 ± 0.26	0.79	0.86	0.76 ± 0.26	0.73	0.80
	SAH	0.83 ± 0.34	0.79	0.86	0.77 ± 0.31	0.73	0.80	0.70 ± 0.30	0.67	0.74
	SDH	0.80 ± 0.37	0.76	0.83	0.74 ± 0.35	0.71	0.77	0.68 ± 0.33	0.65	0.71
	EDH	0.91 ± 0.26	0.82	1.01	0.84 ± 0.24	0.75	0.92	0.77 ± 0.24	0.68	0.85
YOLOV8 large	IVH	0.73 ± 0.42	0.60	0.86	0.64 ± 0.37	0.53	0.76	0.57 ± 0.33	0.46	0.67
	IPH	0.87 ± 0.30	0.83	0.90	0.80 ± 0.28	0.76	0.83	0.73 ± 0.27	0.70	0.77
	SAH	0.75 ± 0.38	0.71	0.79	0.69 ± 0.35	0.65	0.73	0.62 ± 0.32	0.59	0.66
	SDH	0.81 ± 0.35	0.78	0.84	0.75 ± 0.32	0.72	0.78	0.68 ± 0.31	0.66	0.71
	EDH	0.89 ± 0.30	0.79	1.00	0.82 ± 0.28	0.71	0.92	0.75 ± 0.27	0.65	0.85
RetinaNet	IVH	0.61 ± 0.44	0.47	0.74	0.55 ± 0.37	0.44	0.66	0.47 ± 0.31	0.37	0.56
	IPH	0.79 ± 0.34	0.75	0.83	0.76 ± 0.27	0.73	0.79	0.67 ± 0.25	0.64	0.70
	SAH	0.62 ± 0.40	0.57	0.66	0.70 ± 0.29	0.67	0.73	0.61 ± 0.27	0.58	0.63
	SDH	0.71 ± 0.35	0.67	0.74	0.77 ± 0.21	0.75	0.79	0.67 ± 0.21	0.65	0.69
	EDH	0.83 ± 0.35	0.70	0.96	0.76 ± 0.31	0.65	0.87	0.68 ± 0.30	0.57	0.79
FRCNN (ResNet101)	IVH	**0.94 ± 0.21**	0.87	1.00	**0.86 ± 0.21**	0.79	0.92	0.79 ± 0.21	0.73	0.86
	IPH	**0.94 ± 0.21**	0.91	0.96	**0.88 ± 0.21**	0.86	0.91	0.83 ± 0.21	0.80	0.86
	SAH	**0.90 ± 0.26**	0.87	0.93	**0.84 ± 0.24**	0.82	0.87	0.78 ± 0.24	0.76	0.81
	SDH	0.94 ± 0.19	0.92	0.96	0.89 ± 0.18	0.88	0.91	0.84 ± 0.18	0.82	0.86
	EDH	0.91 ± 0.26	0.82	1.01	**0.88 ± 0.24**	0.79	0.97	0.83 ± 0.23	0.74	0.92
FRCNN (ResNeXt101)	IVH	0.92 ± 0.26	0.84	0.99	**0.86 ± 0.26**	0.78	0.94	**0.81 ± 0.25**	0.73	0.89
	IPH	0.92 ± 0.24	0.89	0.95	0.88 ± 0.24	0.85	0.91	**0.84 ± 0.24**	0.81	0.87
	SAH	0.88 ± 0.30	0.84	0.91	0.83 ± 0.29	0.80	0.86	**0.79 ± 0.28**	0.76	0.82
	SDH	0.94 ± 0.21	0.92	0.95	**0.90 ± 0.20**	0.88	0.92	**0.86 ± 0.20**	0.84	0.88
	EDH	0.93 ± 0.25	0.84	1.02	**0.88 ± 0.24**	0.79	0.97	**0.84 ± 0.25**	0.75	0.93
Swin-RT-DETR	IVH	0.90 ± 0.26	0.82	0.98	0.84 ± 0.21	0.77	0.90	0.76 ± 0.21	0.70	0.83
	IPH	**0.94 ± 0.22**	0.91	0.96	**0.88 ± 0.21**	0.85	0.90	0.82 ± 0.21	0.80	0.85
	SAH	**0.90 ± 0.27**	0.87	0.92	**0.84 ± 0.25**	0.81	0.86	0.78 ± 0.24	0.75	0.80
	SDH	**0.95 ± 0.18**	0.93	0.96	0.89 ± 0.17	0.87	0.91	0.83 ± 0.18	0.82	0.85
	EDH	**0.96 ± 0.18**	0.89	1.02	**0.88 ± 0.18**	0.81	0.94	0.81 ± 0.20	0.74	0.88

Note: mAP@50 denotes mean average precision at a bounding-box intersection of union threshold of 0.5; BB-DSC denotes Bounding-box Dice Similarity Coefficient; BB-IoU denotes bounding-box intersection over union; ICH denotes intracranial hemorrhage; IPH denotes intraparenchymal hemorrhage; IVH denotes intraventricular hemorrhage; SAH denotes subarachnoid hemorrhage; EDH denotes epidural hemorrhage.

Table S4. Image-level Quantitative Results of Deep Learning Models Trained on BHX Dataset and Tested on RSNA+ Dataset.

Model	ICH Subtypes	mAP@50			BB-DSC			BB-IoU		
		Mean ± Std	95% Confidence Interval		Mean ± Std	95% Confidence Interval		Mean ± Std	95% Confidence Interval	
			Lower Bound	Upper Bound		Lower Bound	Upper Bound		Lower Bound	Upper Bound
YOLOV8 small	IVH	0.12 ± 0.30	0.10	0.13	0.08 ± 0.21	0.07	0.09	0.06 ± 0.17	0.06	0.07
	IPH	0.47 ± 0.49	0.45	0.48	0.37 ± 0.39	0.36	0.38	0.30 ± 0.32	0.29	0.32
	SAH	0.07 ± 0.24	0.06	0.08	0.06 ± 0.20	0.05	0.07	0.05 ± 0.16	0.04	0.05
	SDH	0.32 ± 0.44	0.31	0.34	0.28 ± 0.37	0.27	0.29	0.24 ± 0.32	0.22	0.25
	EDH	0.20 ± 0.39	0.13	0.27	0.16 ± 0.31	0.10	0.21	0.13 ± 0.26	0.09	0.18
YOLOV8 large	IVH	0.17 ± 0.35	0.15	0.18	0.12 ± 0.25	0.11	0.13	0.10 ± 0.20	0.09	0.10
	IPH	0.44 ± 0.48	0.42	0.45	0.34 ± 0.38	0.33	0.36	0.28 ± 0.32	0.27	0.29
	SAH	0.08 ± 0.24	0.07	0.09	0.07 ± 0.20	0.06	0.08	0.05 ± 0.16	0.05	0.06
	SDH	**0.41 ± 0.45**	0.39	0.42	0.37 ± 0.39	0.35	0.38	0.31 ± 0.33	0.30	0.32
	EDH	0.28 ± 0.44	0.20	0.35	0.22 ± 0.36	0.16	0.28	0.18 ± 0.30	0.13	0.24
RetinaNet	IVH	**0.27 ± 0.36**	0.25	0.28	**0.28 ± 0.34**	0.26	0.29	**0.23 ± 0.28**	0.21	0.24
	IPH	**0.48 ± 0.44**	0.47	0.50	**0.51 ± 0.37**	0.50	0.53	**0.42 ± 0.31**	0.41	0.44
	SAH	**0.12 ± 0.25**	0.11	0.13	**0.22 ± 0.32**	0.21	0.24	**0.18 ± 0.26**	0.17	0.19
	SDH	0.38 ± 0.39	0.37	0.39	**0.57 ± 0.34**	0.55	0.58	**0.47 ± 0.29**	0.46	0.48
	EDH	**0.44 ± 0.48**	0.36	0.53	**0.38 ± 0.40**	0.31	0.45	**0.32 ± 0.34**	0.26	0.38
FRCNN (ResNet101)	IVH	0.24 ± 0.39	0.22	0.25	0.17 ± 0.29	0.16	0.18	0.14 ± 0.24	0.13	0.15
	IPH	0.47 ± 0.49	0.45	0.48	0.37 ± 0.39	0.35	0.38	0.30 ± 0.32	0.29	0.31
	SAH	0.07 ± 0.25	0.06	0.08	0.06 ± 0.19	0.05	0.07	0.05 ± 0.16	0.04	0.05
	SDH	0.37 ± 0.45	0.36	0.39	0.32 ± 0.38	0.31	0.33	0.27 ± 0.32	0.26	0.28
	EDH	0.27 ± 0.44	0.19	0.35	0.22 ± 0.36	0.16	0.28	0.18 ± 0.31	0.13	0.24
FRCNN (ResNeXt101)	IVH	0.23 ± 0.40	0.22	0.25	0.17 ± 0.30	0.15	0.18	0.14 ± 0.24	0.13	0.15
	IPH	0.41 ± 0.48	0.39	0.43	0.32 ± 0.38	0.31	0.33	0.26 ± 0.32	0.25	0.28
	SAH	0.06 ± 0.23	0.05	0.07	0.05 ± 0.18	0.04	0.05	0.04 ± 0.14	0.03	0.04
	SDH	0.34 ± 0.45	0.33	0.36	0.29 ± 0.38	0.27	0.30	0.24 ± 0.32	0.23	0.25
	EDH	0.16 ± 0.37	0.10	0.23	0.13 ± 0.30	0.08	0.18	0.11 ± 0.25	0.07	0.15
Swin-RT-DETR	IVH	0.20 ± 0.37	0.18	0.21	0.14 ± 0.27	0.13	0.16	0.12 ± 0.22	0.11	0.13
	IPH	0.43 ± 0.49	0.41	0.45	0.34 ± 0.38	0.32	0.35	0.28 ± 0.32	0.27	0.29
	SAH	0.07 ± 0.24	0.06	0.08	0.05 ± 0.18	0.04	0.06	0.04 ± 0.15	0.03	0.05
	SDH	0.39 ± 0.46	0.37	0.40	0.32 ± 0.39	0.31	0.34	0.27 ± 0.33	0.26	0.29
	EDH	0.12 ± 0.33	0.07	0.18	0.10 ± 0.26	0.05	0.14	0.08 ± 0.22	0.04	0.12

Note: mAP@50 denotes mean average precision at a bounding-box intersection of union threshold of 0.5; BB-DSC denotes Bounding-box Dice Similarity Coefficient; BB-IoU denotes bounding-box intersection over union; ICH denotes intracranial hemorrhage; IPH denotes intraparenchymal hemorrhage; IVH denotes intraventricular hemorrhage; SAH denotes subarachnoid hemorrhage; EDH denotes epidural hemorrhage.

Table S5. Image-level Quantitative Results of Deep Learning Models Trained on RSNA+ Dataset and Tested on RSNA+ Dataset.

Model	ICH Subtypes	mAP@50 Mean ± Std	mAP@50 95% CI Lower Bound	mAP@50 95% CI Upper Bound	BB-DSC Mean ± Std	BB-DSC 95% CI Lower Bound	BB-DSC 95% CI Upper Bound	BB-IoU Mean ± Std	BB-IoU 95% CI Lower Bound	BB-IoU 95% CI Upper Bound
YOLOV8 small	IVH	**0.95 ± 0.18**	0.92	0.97	**0.88 ± 0.18**	0.86	0.90	**0.82 ± 0.18**	0.79	0.84
	IPH	0.90 ± 0.27	0.87	0.93	0.86 ± 0.26	0.83	0.89	0.81 ± 0.26	0.78	0.84
	SAH	0.85 ± 0.30	0.80	0.89	0.79 ± 0.27	0.75	0.83	**0.72 ± 0.26**	0.69	0.76
	SDH	0.90 ± 0.25	0.87	0.93	0.85 ± 0.22	0.83	0.88	0.79 ± 0.23	0.77	0.82
	EDH	0.76 ± 0.43	0.50	1.02	0.72 ± 0.42	0.47	0.97	0.69 ± 0.40	0.44	0.93
YOLOV8 large	IVH	0.94 ± 0.18	0.92	0.97	0.87 ± 0.18	0.85	0.90	0.81 ± 0.19	0.78	0.83
	IPH	0.90 ± 0.28	0.87	0.93	0.85 ± 0.27	0.82	0.88	0.80 ± 0.26	0.77	0.83
	SAH	0.82 ± 0.32	0.78	0.87	0.75 ± 0.30	0.71	0.80	0.69 ± 0.29	0.65	0.73
	SDH	0.87 ± 0.30	0.84	0.90	0.83 ± 0.28	0.80	0.86	0.77 ± 0.27	0.74	0.80
	EDH	0.61 ± 0.50	0.31	0.91	0.58 ± 0.48	0.29	0.87	0.55 ± 0.46	0.27	0.83
RetinaNet	IVH	0.92 ± 0.20	0.90	0.95	0.85 ± 0.19	0.83	0.88	0.78 ± 0.19	0.76	0.81
	IPH	0.89 ± 0.29	0.86	0.92	0.84 ± 0.27	0.81	0.87	0.78 ± 0.26	0.75	0.81
	SAH	0.81 ± 0.32	0.76	0.85	0.76 ± 0.27	0.72	0.80	0.68 ± 0.25	0.64	0.72
	SDH	0.86 ± 0.29	0.83	0.90	0.82 ± 0.25	0.80	0.85	0.75 ± 0.24	0.72	0.77
	EDH	**0.84 ± 0.37**	0.61	1.06	**0.79 ± 0.35**	0.58	1.00	**0.74 ± 0.33**	0.54	0.95
FRCNN (ResNet101)	IVH	0.90 ± 0.26	0.87	0.94	0.82 ± 0.24	0.79	0.86	0.76 ± 0.23	0.73	0.79
	IPH	0.89 ± 0.28	0.86	0.93	0.84 ± 0.27	0.80	0.87	0.78 ± 0.27	0.75	0.81
	SAH	0.77 ± 0.36	0.71	0.82	0.70 ± 0.32	0.65	0.74	0.63 ± 0.30	0.58	0.67
	SDH	0.85 ± 0.32	0.82	0.88	0.80 ± 0.29	0.77	0.83	0.73 ± 0.28	0.70	0.76
	EDH	**0.84 ± 0.37**	0.61	1.06	0.78 ± 0.35	0.57	0.99	0.73 ± 0.33	0.52	0.93
FRCNN (ResNeXt101)	IVH	0.90 ± 0.25	0.87	0.94	0.82 ± 0.24	0.78	0.85	0.74 ± 0.24	0.71	0.78
	IPH	0.90 ± 0.27	0.87	0.93	0.84 ± 0.26	0.81	0.87	0.79 ± 0.26	0.76	0.82
	SAH	0.77 ± 0.36	0.72	0.82	0.69 ± 0.33	0.64	0.74	0.62 ± 0.31	0.58	0.66
	SDH	0.86 ± 0.31	0.83	0.89	0.81 ± 0.28	0.78	0.84	0.75 ± 0.27	0.72	0.77
	EDH	0.76 ± 0.43	0.50	1.02	0.70 ± 0.40	0.46	0.95	0.65 ± 0.38	0.42	0.88
Swin-RT-DETR	IVH	0.93 ± 0.21	0.90	0.95	0.86 ± 0.18	0.83	0.88	0.78 ± 0.18	0.76	0.81
	IPH	**0.91 ± 0.25**	0.88	0.94	**0.86 ± 0.23**	0.84	0.89	**0.81 ± 0.23**	0.78	0.83
	SAH	**0.86 ± 0.28**	0.82	0.90	**0.79 ± 0.24**	0.76	0.83	0.70 ± 0.22	0.67	0.74
	SDH	**0.91 ± 0.22**	0.88	0.93	**0.88 ± 0.16**	0.86	0.90	**0.81 ± 0.17**	0.80	0.83
	EDH	0.38 ± 0.50	0.08	0.68	0.36 ± 0.47	0.07	0.65	0.34 ± 0.45	0.07	0.61

Note: mAP@50 denotes mean average precision at a bounding-box intersection of union threshold of 0.5; BB-DSC denotes Bounding-box Dice Similarity Coefficient; BB-IoU denotes bounding-box intersection over union; ICH denotes intracranial hemorrhage; IPH denotes intraparenchymal hemorrhage; IVH denotes intraventricular hemorrhage; SAH denotes subarachnoid hemorrhage; EDH denotes epidural hemorrhage.

Table S6. Image-level Quantitative Results of Deep Learning Models Trained on RSNA+ Dataset and Tested on BHX Dataset.

Model	ICH Subtypes	mAP@50			BB-DSC			BB-IoU		
		Mean ± Std	95% Confidence Interval		Mean ± Std	95% Confidence Interval		Mean ± Std	95% Confidence Interval	
			Lower Bound	Upper Bound		Lower Bound	Upper Bound		Lower Bound	Upper Bound
YOLOV8 small	IVH	0.26 ± 0.42	0.22	0.30	0.19 ± 0.32	0.16	0.22	0.15 ± 0.26	0.13	0.18
	IPH	0.34 ± 0.47	0.33	0.36	0.28 ± 0.38	0.26	0.29	0.23 ± 0.32	0.22	0.24
	SAH	0.05 ± 0.21	0.04	0.06	0.04 ± 0.16	0.03	0.04	0.03 ± 0.13	0.03	0.04
	SDH	0.31 ± 0.44	0.30	0.32	0.26 ± 0.37	0.25	0.27	0.22 ± 0.32	0.22	0.23
	EDH	0.17 ± 0.38	0.13	0.21	0.15 ± 0.32	0.11	0.18	0.13 ± 0.29	0.10	0.16
YOLOV8 large	IVH	0.26 ± 0.42	0.22	0.30	0.19 ± 0.31	0.16	0.22	0.15 ± 0.25	0.13	0.18
	IPH	0.34 ± 0.47	0.32	0.35	0.27 ± 0.37	0.25	0.28	0.22 ± 0.31	0.21	0.23
	SAH	0.05 ± 0.21	0.04	0.06	0.04 ± 0.17	0.03	0.05	0.03 ± 0.14	0.03	0.04
	SDH	0.31 ± 0.44	0.30	0.32	0.26 ± 0.37	0.25	0.27	0.22 ± 0.32	0.21	0.23
	EDH	0.16 ± 0.36	0.12	0.20	0.14 ± 0.32	0.10	0.17	0.12 ± 0.28	0.09	0.15
RetinaNet	IVH	0.32 ± 0.44	0.28	0.36	0.26 ± 0.35	0.22	0.29	0.21 ± 0.28	0.18	0.23
	IPH	0.33 ± 0.46	0.31	0.35	0.27 ± 0.37	0.26	0.29	0.22 ± 0.31	0.21	0.24
	SAH	0.05 ± 0.20	0.05	0.06	0.05 ± 0.18	0.04	0.06	0.04 ± 0.15	0.03	0.04
	SDH	0.31 ± 0.42	0.30	0.33	0.28 ± 0.37	0.27	0.29	0.23 ± 0.31	0.23	0.24
	EDH	**0.33 ± 0.47**	0.28	0.38	**0.28 ± 0.39**	0.23	0.32	**0.24 ± 0.33**	0.20	0.27
FRCNN (ResNet101)	IVH	**0.33 ± 0.45**	0.29	0.37	0.25 ± 0.34	0.22	0.28	0.20 ± 0.28	0.18	0.23
	IPH	0.36 ± 0.47	0.34	0.38	0.29 ± 0.38	0.27	0.30	0.24 ± 0.32	0.23	0.25
	SAH	0.05 ± 0.21	0.05	0.06	0.04 ± 0.17	0.04	0.05	0.03 ± 0.14	0.03	0.04
	SDH	0.29 ± 0.43	0.28	0.30	0.24 ± 0.36	0.23	0.25	0.20 ± 0.31	0.20	0.21
	EDH	0.23 ± 0.42	0.18	0.28	0.19 ± 0.36	0.15	0.23	0.17 ± 0.31	0.13	0.20
FRCNN (ResNeXt101)	IVH	0.33 ± 0.45	0.28	0.37	0.25 ± 0.34	0.22	0.28	0.20 ± 0.28	0.17	0.23
	IPH	0.36 ± 0.47	0.34	0.38	0.29 ± 0.38	0.27	0.30	0.24 ± 0.32	0.23	0.25
	SAH	0.05 ± 0.21	0.04	0.06	0.04 ± 0.16	0.03	0.04	0.03 ± 0.13	0.03	0.03
	SDH	0.29 ± 0.43	0.28	0.30	0.24 ± 0.36	0.23	0.25	0.21 ± 0.31	0.20	0.22
	EDH	0.20 ± 0.40	0.15	0.24	0.16 ± 0.33	0.13	0.20	0.14 ± 0.28	0.11	0.17
Swin-RT-DETR	IVH	0.32 ± 0.44	0.28	0.37	**0.26 ± 0.35**	0.23	0.30	**0.21 ± 0.28**	0.19	0.24
	IPH	**0.38 ± 0.47**	0.36	0.39	**0.31 ± 0.38**	0.29	0.32	**0.25 ± 0.32**	0.24	0.27
	SAH	**0.06 ± 0.23**	0.05	0.07	**0.05 ± 0.19**	0.05	0.06	**0.04 ± 0.15**	0.04	0.05
	SDH	**0.35 ± 0.44**	0.33	0.36	**0.32 ± 0.38**	0.31	0.33	**0.27 ± 0.33**	0.26	0.28
	EDH	0.23 ± 0.42	0.18	0.27	0.19 ± 0.36	0.15	0.23	0.17 ± 0.31	0.13	0.20

Note: mAP@50 denotes mean average precision at a bounding-box intersection of union threshold of 0.5; BB-DSC denotes Bounding-box Dice Similarity Coefficient; BB-IoU denotes bounding-box intersection over union; ICH denotes intracranial hemorrhage; IPH denotes intraparenchymal hemorrhage; IVH denotes intraventricular hemorrhage; SAH denotes subarachnoid hemorrhage; EDH denotes epidural hemorrhage.

Table S7. Patient-level Quantitative Results of Deep Learning Models Trained on BHX Dataset and Tested on BHX Dataset.

Model	ICH Subtypes	mAP@50			BB-DSC			BB-IoU		
		Mean ± Std	95% Confidence Interval		Mean ± Std	95% Confidence Interval		Mean ± Std	95% Confidence Interval	
			Lower Bound	Upper Bound		Lower Bound	Upper Bound		Lower Bound	Upper Bound
YOLOV8 small	IVH	0.80 ± 0.32	0.61	0.99	0.74 ± 0.29	0.56	0.92	0.67 ± 0.27	0.51	0.84
	IPH	0.88 ± 0.22	0.83	0.93	0.79 ± 0.22	0.74	0.85	0.73 ± 0.22	0.68	0.78
	SAH	0.83 ± 0.34	0.79	0.86	0.77 ± 0.31	0.73	0.80	0.70 ± 0.30	0.67	0.74
	SDH	0.71 ± 0.36	0.63	0.80	0.64 ± 0.33	0.57	0.72	0.58 ± 0.31	0.51	0.65
	EDH	0.80 ± 0.39	0.38	1.21	0.73 ± 0.36	0.35	1.11	0.67 ± 0.34	0.32	1.02
YOLOV8 large	IVH	0.68 ± 0.32	0.49	0.88	0.61 ± 0.30	0.43	0.79	0.54 ± 0.28	0.37	0.71
	IPH	0.86 ± 0.24	0.80	0.91	0.78 ± 0.22	0.73	0.83	0.71 ± 0.21	0.66	0.76
	SAH	0.75 ± 0.38	0.71	0.79	0.69 ± 0.35	0.65	0.73	0.62 ± 0.32	0.59	0.66
	SDH	0.64 ± 0.38	0.55	0.72	0.58 ± 0.34	0.50	0.66	0.51 ± 0.31	0.44	0.59
	EDH	0.76 ± 0.39	0.36	1.17	0.70 ± 0.36	0.32	1.07	0.65 ± 0.34	0.29	1.00
RetinaNet	IVH	0.60 ± 0.33	0.40	0.80	0.54 ± 0.28	0.37	0.71	0.46 ± 0.24	0.31	0.60
	IPH	0.76 ± 0.29	0.69	0.83	0.74 ± 0.23	0.69	0.79	0.65 ± 0.21	0.60	0.70
	SAH	0.62 ± 0.40	0.57	0.66	0.70 ± 0.29	0.67	0.73	0.61 ± 0.27	0.58	0.63
	SDH	0.49 ± 0.33	0.41	0.56	0.62 ± 0.27	0.56	0.68	0.53 ± 0.24	0.47	0.59
	EDH	0.72 ± 0.37	0.33	1.11	0.66 ± 0.34	0.30	1.02	0.60 ± 0.32	0.26	0.93
FRCNN (ResNet101)	IVH	**0.90 ± 0.27**	0.73	1.06	**0.82 ± 0.26**	0.66	0.97	0.75 ± 0.25	0.60	0.90
	IPH	**0.91 ± 0.21**	0.86	0.96	**0.85 ± 0.21**	0.80	0.90	0.79 ± 0.20	0.74	0.84
	SAH	**0.90 ± 0.26**	0.87	0.93	**0.84 ± 0.24**	0.82	0.87	0.78 ± 0.24	0.76	0.81
	SDH	0.77 ± 0.36	0.69	0.86	0.72 ± 0.33	0.64	0.80	0.66 ± 0.31	0.59	0.73
	EDH	0.80 ± 0.39	0.38	1.21	**0.77 ± 0.38**	0.37	1.16	**0.73 ± 0.36**	0.35	1.10
FRCNN (ResNeXt101)	IVH	0.85 ± 0.29	0.67	1.03	0.80 ± 0.29	0.63	0.97	**0.76 ± 0.28**	0.59	0.93
	IPH	0.89 ± 0.23	0.83	0.95	0.84 ± 0.24	0.78	0.90	**0.80 ± 0.23**	0.74	0.85
	SAH	0.88 ± 0.30	0.84	0.91	0.83 ± 0.29	0.80	0.86	**0.79 ± 0.28**	0.76	0.82
	SDH	0.75 ± 0.36	0.67	0.84	0.70 ± 0.35	0.62	0.78	0.65 ± 0.33	0.58	0.73
	EDH	0.80 ± 0.40	0.39	1.22	**0.77 ± 0.38**	0.37	1.17	**0.73 ± 0.37**	0.35	1.12
Swin-RT-DETR	IVH	0.87 ± 0.27	0.71	1.04	0.81 ± 0.25	0.65	0.96	0.74 ± 0.24	0.59	0.88
	IPH	**0.91 ± 0.21**	0.86	0.96	**0.85 ± 0.20**	0.80	0.90	0.79 ± 0.19	0.74	0.83
	SAH	**0.90 ± 0.27**	0.87	0.92	**0.84 ± 0.25**	0.81	0.86	0.78 ± 0.24	0.75	0.80
	SDH	**0.82 ± 0.31**	0.74	0.89	**0.75 ± 0.29**	0.68	0.81	**0.68 ± 0.27**	0.62	0.75
	EDH	**0.83 ± 0.40**	0.40	1.25	0.76 ± 0.37	0.37	1.15	0.70 ± 0.35	0.34	1.07

Note: mAP@50 denotes mean average precision at a bounding-box intersection of union threshold of 0.5; BB-DSC denotes Bounding-box Dice Similarity Coefficient; BB-IoU denotes bounding-box intersection over union; ICH denotes intracranial hemorrhage; IPH denotes intraparenchymal hemorrhage; IVH denotes intraventricular hemorrhage; SAH denotes subarachnoid hemorrhage; EDH denotes epidural hemorrhage.

Table S8. Patient-level Quantitative Results of Deep Learning Models Trained on BHX Dataset and Tested on RSNA+ Dataset.

Model	ICH Subtypes	mAP@50 Mean ± Std	mAP@50 95% Confidence Interval Lower Bound	mAP@50 95% Confidence Interval Upper Bound	BB-DSC Mean ± Std	BB-DSC 95% Confidence Interval Lower Bound	BB-DSC 95% Confidence Interval Upper Bound	BB-IoU Mean ± Std	BB-IoU 95% Confidence Interval Lower Bound	BB-IoU 95% Confidence Interval Upper Bound
YOLOV8 small	IVH	0.09 ± 0.18	0.08	0.11	0.07 ± 0.12	0.05	0.08	0.05 ± 0.10	0.04	0.06
	IPH	0.39 ± 0.35	0.37	0.42	0.31 ± 0.28	0.29	0.33	0.25 ± 0.23	0.24	0.27
	SAH	0.06 ± 0.14	0.04	0.07	0.05 ± 0.12	0.04	0.06	0.04 ± 0.09	0.03	0.05
	SDH	0.27 ± 0.27	0.24	0.29	0.23 ± 0.23	0.21	0.25	0.19 ± 0.19	0.17	0.21
	EDH	0.16 ± 0.24	0.07	0.26	0.13 ± 0.19	0.06	0.21	0.11 ± 0.15	0.05	0.17
YOLOV8 large	IVH	0.18 ± 0.25	0.15	0.20	0.13 ± 0.18	0.11	0.14	0.10 ± 0.15	0.09	0.11
	IPH	0.36 ± 0.34	0.33	0.38	0.28 ± 0.27	0.26	0.30	0.23 ± 0.22	0.21	0.24
	SAH	0.06 ± 0.14	0.05	0.07	0.05 ± 0.12	0.04	0.06	0.04 ± 0.09	0.03	0.05
	SDH	**0.34 ± 0.30**	0.31	0.37	0.30 ± 0.26	0.28	0.33	0.25 ± 0.22	0.23	0.27
	EDH	0.28 ± 0.35	0.14	0.41	0.22 ± 0.29	0.11	0.33	0.18 ± 0.25	0.09	0.28
RetinaNet	IVH	**0.23 ± 0.25**	0.20	0.25	**0.23 ± 0.23**	0.20	0.25	**0.18 ± 0.18**	0.17	0.20
	IPH	**0.41 ± 0.32**	0.38	0.43	**0.46 ± 0.28**	0.44	0.48	**0.38 ± 0.24**	0.36	0.40
	SAH	**0.10 ± 0.17**	0.08	0.12	**0.19 ± 0.22**	0.17	0.21	**0.15 ± 0.18**	0.14	0.17
	SDH	0.33 ± 0.26	0.31	0.36	**0.53 ± 0.23**	0.51	0.55	**0.43 ± 0.19**	0.42	0.45
	EDH	**0.38 ± 0.38**	0.23	0.53	**0.33 ± 0.34**	0.20	0.47	**0.28 ± 0.29**	0.16	0.39
FRCNN (ResNet101)	IVH	0.20 ± 0.26	0.17	0.22	0.14 ± 0.19	0.12	0.16	0.11 ± 0.16	0.10	0.13
	IPH	0.39 ± 0.35	0.36	0.42	0.30 ± 0.28	0.28	0.32	0.25 ± 0.23	0.23	0.27
	SAH	0.06 ± 0.15	0.04	0.07	0.04 ± 0.11	0.03	0.06	0.04 ± 0.09	0.03	0.04
	SDH	0.30 ± 0.29	0.27	0.33	0.26 ± 0.24	0.23	0.28	0.21 ± 0.20	0.19	0.24
	EDH	0.24 ± 0.34	0.10	0.37	0.19 ± 0.27	0.08	0.29	0.15 ± 0.22	0.07	0.24
FRCNN (ResNeXt101)	IVH	0.19 ± 0.25	0.17	0.21	0.14 ± 0.18	0.12	0.15	0.11 ± 0.15	0.10	0.12
	IPH	0.33 ± 0.33	0.31	0.36	0.26 ± 0.26	0.24	0.28	0.21 ± 0.22	0.20	0.23
	SAH	0.04 ± 0.12	0.03	0.06	0.03 ± 0.09	0.03	0.04	0.03 ± 0.08	0.02	0.04
	SDH	0.26 ± 0.27	0.24	0.29	0.22 ± 0.23	0.20	0.24	0.18 ± 0.19	0.16	0.20
	EDH	0.16 ± 0.28	0.05	0.27	0.12 ± 0.22	0.04	0.21	0.10 ± 0.17	0.03	0.17
Swin-RT-DETR	IVH	0.17 ± 0.24	0.15	0.19	0.13 ± 0.18	0.11	0.14	0.10 ± 0.15	0.09	0.12
	IPH	0.36 ± 0.35	0.33	0.38	0.28 ± 0.28	0.26	0.30	0.23 ± 0.23	0.21	0.25
	SAH	0.05 ± 0.13	0.04	0.06	0.04 ± 0.11	0.03	0.05	0.03 ± 0.09	0.02	0.04
	SDH	0.33 ± 0.29	0.30	0.36	0.27 ± 0.24	0.25	0.30	0.23 ± 0.21	0.21	0.25
	EDH	0.09 ± 0.22	0.00	0.17	0.07 ± 0.16	0.01	0.13	0.05 ± 0.12	0.01	0.10

Note: mAP@50 denotes mean average precision at a bounding-box intersection of union threshold of 0.5; BB-DSC denotes Bounding-box Dice Similarity Coefficient; BB-IoU denotes bounding-box intersection over union; ICH denotes intracranial hemorrhage; IPH denotes intraparenchymal hemorrhage; IVH denotes intraventricular hemorrhage; SAH denotes subarachnoid hemorrhage; EDH denotes epidural hemorrhage.

Table S9. Patient-level Quantitative Results of Deep Learning Models Trained on RSNA+ Dataset and Tested on RSNA+ Dataset.

Model	ICH Subtypes	mAP@50			BB-DSC			BB-IoU		
		Mean ± Std	95% Confidence Interval Lower Bound	95% Confidence Interval Upper Bound	Mean ± Std	95% Confidence Interval Lower Bound	95% Confidence Interval Upper Bound	Mean ± Std	95% Confidence Interval Lower Bound	95% Confidence Interval Upper Bound
YOLOV8 small	IVH	**0.94 ± 0.18**	0.91	0.97	**0.87 ± 0.18**	0.85	0.90	**0.81 ± 0.18**	0.78	0.84
	IPH	0.89 ± 0.27	0.86	0.93	**0.85 ± 0.25**	0.82	0.89	**0.81 ± 0.25**	0.77	0.84
	SAH	**0.85 ± 0.27**	0.80	0.89	**0.79 ± 0.25**	0.75	0.83	**0.72 ± 0.24**	0.68	0.76
	SDH	0.89 ± 0.23	0.86	0.93	0.85 ± 0.21	0.82	0.88	0.79 ± 0.21	0.76	0.81
	EDH	0.72 ± 0.44	0.38	1.05	0.68 ± 0.41	0.36	1.00	0.65 ± 0.39	0.35	0.95
YOLOV8 large	IVH	**0.94 ± 0.19**	0.91	0.97	**0.87 ± 0.19**	0.84	0.90	0.80 ± 0.19	0.78	0.83
	IPH	0.89 ± 0.27	0.86	0.93	0.84 ± 0.26	0.81	0.88	0.80 ± 0.26	0.77	0.83
	SAH	0.82 ± 0.29	0.77	0.87	0.75 ± 0.27	0.71	0.80	0.69 ± 0.26	0.65	0.73
	SDH	0.86 ± 0.29	0.82	0.89	0.81 ± 0.27	0.78	0.85	0.76 ± 0.26	0.73	0.80
	EDH	0.57 ± 0.46	0.21	0.92	0.53 ± 0.43	0.20	0.86	0.50 ± 0.40	0.19	0.81
RetinaNet	IVH	0.92 ± 0.20	0.89	0.95	0.85 ± 0.18	0.82	0.88	0.78 ± 0.18	0.75	0.81
	IPH	0.89 ± 0.26	0.86	0.93	0.84 ± 0.24	0.81	0.87	0.78 ± 0.23	0.75	0.81
	SAH	0.81 ± 0.28	0.77	0.86	0.76 ± 0.24	0.72	0.80	0.68 ± 0.23	0.64	0.72
	SDH	0.85 ± 0.28	0.81	0.89	0.81 ± 0.24	0.78	0.84	0.74 ± 0.24	0.70	0.77
	EDH	**0.90 ± 0.19**	0.76	1.04	**0.85 ± 0.17**	0.72	0.98	**0.79 ± 0.16**	0.68	0.91
FRCNN (ResNet101)	IVH	0.90 ± 0.24	0.86	0.94	0.82 ± 0.23	0.79	0.86	0.76 ± 0.22	0.72	0.79
	IPH	0.89 ± 0.27	0.86	0.93	0.83 ± 0.26	0.80	0.87	0.78 ± 0.26	0.74	0.81
	SAH	0.78 ± 0.32	0.72	0.83	0.70 ± 0.29	0.65	0.75	0.63 ± 0.27	0.58	0.67
	SDH	0.83 ± 0.31	0.79	0.87	0.78 ± 0.28	0.74	0.82	0.72 ± 0.27	0.68	0.75
	EDH	0.83 ± 0.35	0.56	1.09	0.77 ± 0.33	0.52	1.02	0.72 ± 0.31	0.48	0.96
FRCNN (ResNeXt101)	IVH	0.90 ± 0.25	0.86	0.94	0.81 ± 0.24	0.78	0.85	0.74 ± 0.23	0.71	0.78
	IPH	**0.90 ± 0.27**	0.86	0.93	0.84 ± 0.25	0.80	0.87	0.78 ± 0.25	0.75	0.81
	SAH	0.78 ± 0.33	0.72	0.84	0.69 ± 0.30	0.64	0.74	0.62 ± 0.28	0.58	0.67
	SDH	0.84 ± 0.31	0.80	0.88	0.79 ± 0.28	0.75	0.82	0.73 ± 0.27	0.69	0.76
	EDH	0.72 ± 0.44	0.38	1.05	0.67 ± 0.41	0.35	0.98	0.62 ± 0.38	0.33	0.91
Swin-RT-DETR	IVH	0.92 ± 0.21	0.89	0.95	0.85 ± 0.19	0.82	0.88	0.78 ± 0.19	0.75	0.80
	IPH	**0.90 ± 0.26**	0.87	0.93	**0.85 ± 0.24**	0.82	0.88	0.80 ± 0.23	0.77	0.83
	SAH	**0.85 ± 0.28**	0.80	0.90	0.78 ± 0.23	0.75	0.82	0.70 ± 0.22	0.66	0.73
	SDH	**0.90 ± 0.20**	0.88	0.93	**0.88 ± 0.15**	0.86	0.90	**0.81 ± 0.16**	0.79	0.83
	EDH	0.40 ± 0.49	0.03	0.78	0.38 ± 0.46	0.03	0.74	0.36 ± 0.43	0.02	0.69

Note: mAP@50 denotes mean average precision at a bounding-box intersection of union threshold of 0.5; BB-DSC denotes Bounding-box Dice Similarity Coefficient; BB-IoU denotes bounding-box intersection over union; ICH denotes intracranial hemorrhage; IPH denotes intraparenchymal hemorrhage; IVH denotes intraventricular hemorrhage; SAH denotes subarachnoid hemorrhage; EDH denotes epidural hemorrhage.

Table S10. Patient-level Quantitative Results of Deep Learning Models Trained on RSNA+ Dataset and Tested on BHX Dataset.

Model	ICH Subtypes	mAP@50			BB-DSC			BB-IoU		
		Mean ± Std	95% Confidence Interval		Mean ± Std	95% Confidence Interval		Mean ± Std	95% Confidence Interval	
			Lower Bound	Upper Bound		Lower Bound	Upper Bound		Lower Bound	Upper Bound
YOLOV8 small	IVH	0.23 ± 0.27	0.11	0.36	0.18 ± 0.20	0.08	0.27	0.14 ± 0.16	0.07	0.22
	IPH	0.31 ± 0.34	0.24	0.37	0.24 ± 0.27	0.18	0.29	0.20 ± 0.23	0.15	0.24
	SAH	0.04 ± 0.09	0.02	0.06	0.03 ± 0.07	0.02	0.05	0.03 ± 0.06	0.02	0.04
	SDH	0.24 ± 0.27	0.18	0.30	0.20 ± 0.23	0.15	0.26	0.17 ± 0.20	0.13	0.22
	EDH	0.13 ± 0.20	-0.08	0.35	0.11 ± 0.18	-0.08	0.30	0.10 ± 0.16	-0.07	0.27
YOLOV8 large	IVH	0.23 ± 0.26	0.11	0.35	0.17 ± 0.18	0.08	0.25	0.13 ± 0.14	0.07	0.20
	IPH	0.31 ± 0.34	0.24	0.38	0.24 ± 0.27	0.19	0.29	0.20 ± 0.22	0.15	0.24
	SAH	0.04 ± 0.11	0.02	0.07	0.03 ± 0.08	0.02	0.05	0.03 ± 0.06	0.02	0.04
	SDH	0.24 ± 0.28	0.18	0.30	0.20 ± 0.24	0.15	0.25	0.17 ± 0.21	0.13	0.22
	EDH	0.12 ± 0.21	-0.10	0.34	0.10 ± 0.19	-0.10	0.30	0.09 ± 0.17	-0.09	0.27
RetinaNet	IVH	**0.32 ± 0.32**	0.17	0.47	0.25 ± 0.23	0.14	0.35	0.19 ± 0.18	0.11	0.28
	IPH	0.31 ± 0.34	0.24	0.38	0.25 ± 0.27	0.20	0.31	0.21 ± 0.22	0.16	0.25
	SAH	0.06 ± 0.11	0.03	0.08	**0.06 ± 0.11**	0.04	0.08	**0.05 ± 0.09**	0.03	0.06
	SDH	0.27 ± 0.29	0.20	0.33	0.23 ± 0.24	0.18	0.29	0.19 ± 0.21	0.15	0.24
	EDH	**0.25 ± 0.35**	-0.12	0.63	**0.21 ± 0.30**	-0.10	0.52	**0.18 ± 0.25**	-0.08	0.44
FRCNN (ResNet101)	IVH	0.29 ± 0.28	0.16	0.41	0.22 ± 0.23	0.11	0.32	0.18 ± 0.20	0.09	0.27
	IPH	0.33 ± 0.32	0.26	0.39	0.26 ± 0.26	0.20	0.31	0.21 ± 0.22	0.17	0.25
	SAH	0.04 ± 0.10	0.02	0.06	0.03 ± 0.08	0.02	0.05	0.03 ± 0.06	0.01	0.04
	SDH	0.23 ± 0.26	0.17	0.29	0.20 ± 0.23	0.14	0.25	0.17 ± 0.20	0.12	0.21
	EDH	0.17 ± 0.26	-0.10	0.44	0.14 ± 0.22	-0.09	0.37	0.13 ± 0.19	-0.08	0.33
FRCNN (ResNeXt101)	IVH	0.28 ± 0.29	0.15	0.41	0.21 ± 0.22	0.11	0.31	0.17 ± 0.18	0.09	0.25
	IPH	**0.34 ± 0.34**	0.27	0.41	0.26 ± 0.27	0.21	0.32	0.22 ± 0.22	0.17	0.26
	SAH	0.04 ± 0.10	0.02	0.06	0.03 ± 0.08	0.02	0.05	0.03 ± 0.07	0.01	0.04
	SDH	0.23 ± 0.27	0.17	0.29	0.19 ± 0.23	0.14	0.24	0.16 ± 0.20	0.12	0.21
	EDH	0.15 ± 0.24	-0.10	0.39	0.12 ± 0.20	-0.09	0.33	0.10 ± 0.17	-0.08	0.28
Swin-RT-DETR	IVH	**0.32 ± 0.32**	0.18	0.47	**0.26 ± 0.25**	0.14	0.37	**0.21 ± 0.20**	0.12	0.30
	IPH	**0.34 ± 0.33**	0.27	0.41	**0.27 ± 0.27**	0.22	0.33	**0.22 ± 0.22**	0.18	0.27
	SAH	**0.05 ± 0.11**	0.03	0.07	0.05 ± 0.09	0.03	0.07	0.04 ± 0.08	0.02	0.05
	SDH	**0.29 ± 0.28**	0.22	0.35	**0.26 ± 0.24**	0.20	0.31	**0.22 ± 0.21**	0.17	0.27
	EDH	0.17 ± 0.23	-0.07	0.41	0.14 ± 0.20	-0.07	0.35	0.12 ± 0.18	-0.06	0.31

Note: mAP@50 denotes mean average precision at a bounding-box intersection of union threshold of 0.5; BB-DSC denotes Bounding-box Dice Similarity Coefficient; BB-IoU denotes bounding-box intersection over union; ICH denotes intracranial hemorrhage; IPH denotes intraparenchymal hemorrhage; IVH denotes intraventricular hemorrhage; SAH denotes subarachnoid hemorrhage; EDH denotes epidural hemorrhage.

References

1. Liu, J., et al., *Deep learning-based identification and localization of intracranial hemorrhage in patients using a large annotated head computed tomography dataset: A retrospective multicenter study.* Intelligent Medicine, 2025. **5**(1): p. 14-22.
2. Flanders, A.E., et al., *Construction of a Machine Learning Dataset through Collaboration: The RSNA 2019 Brain CT Hemorrhage Challenge.* Radiol Artif Intell, 2020. **2**(3): p. e190211.
3. Reis, E.P., et al., *Brain Hemorrhage Extended (BHX): Bounding box extrapolation from thick to thin slice CT images*. 2020, PhysioNet.
4. Jocher, G., A. Chaurasia, and J. Qiu, *Ultralytics YOLOv8*. 2023, Ultralytics.
5. Lin, T.Y., et al. *Focal Loss for Dense Object Detection*. in *2017 IEEE International Conference on Computer Vision (ICCV)*. 2017.
6. Ren, S., et al., *Faster R-CNN: Towards Real-Time Object Detection with Region Proposal Networks.* IEEE Trans Pattern Anal Mach Intell, 2017. **39**(6): p. 1137-1149.
7. He, K., et al. *Deep Residual Learning for Image Recognition*. in *2016 IEEE Conference on Computer Vision and Pattern Recognition (CVPR)*. 2016.
8. Xie, S., et al. *Aggregated Residual Transformations for Deep Neural Networks*. in *2017 IEEE Conference on Computer Vision and Pattern Recognition (CVPR)*. 2017.

Disclaimer/Publisher's Note: The statements, opinions and data contained in all publications are solely those of the individual author(s) and contributor(s) and not of MDPI and/or the editor(s). MDPI and/or the editor(s) disclaim responsibility for any injury to people or property resulting from any ideas, methods, instructions or products referred to in the content.
